# Mkx mediates tenogenic differentiation but incompletely inhibits the proliferation of hypoxic MSCs

**DOI:** 10.1186/s13287-021-02506-3

**Published:** 2021-07-28

**Authors:** Guanyin Chen, Dong Fan, Wangqian Zhang, Shuning Wang, Jintao Gu, Yuan Gao, Lei He, Weina Li, Cun Zhang, Meng Li, Yingqi Zhang, Zhaohui Liu, Qiang Hao

**Affiliations:** 1 State Key Laboratory of Cancer Biology, Biotechnology Center, School of Pharmacy, Fourth Military Medical University, Xi’an, China; 2 Department of General Surgery, Tangdu Hospital, Fourth Military Medical University, Xi’an, China; 3 Department of Rehabilitation and Physiotherapy, Tangdu Hospital, Fourth Military Medical University, Xi’an, China

**Keywords:** Mkx, Tenegenic differentiation, Proliferation, Hypoxia, Mesenchymal stem cells

## Abstract

**Background:**

Hypoxia has been shown to be able to induce tenogenic differentiation and proliferation of mesenchymal stem cells (MSCs) which lead hypoxia-induced MSCs to be a potential treatment for tendon injury. However, little is known about the mechanism underlying the tenogenic differentiation and proliferation process of hypoxic MSCs, which limited the application of differentiation-inducing therapies in tendon repair. This study was designed to investigate the role of Mohawk homeobox (Mkx) in tenogenic differentiation and proliferation of hypoxic MSCs.

**Methods:**

qRT-PCR, western blot, and immunofluorescence staining were performed to evaluate the expression of Mkx and other tendon-associated markers in adipose-derived MSCs (AMSCs) and bone marrow-derived MSCs (BMSCs) under hypoxia condition. Small interfering RNA technique was applied to observe the effect of Mkx levels on the expression of tendon-associated markers in normoxic and hypoxic BMSCs. Hypoxic BMSCs infected with Mkx-specific short hair RNA (shRNA) or scramble were implanted into the wound gaps of injured patellar tendons to assess the effect of Mkx levels on tendon repair. In addition, cell counting kit-8 assay, colony formation unit assay, cell cycle analysis, and EdU assay were adopted to determine the proliferation capacity of normoxic or hypoxic BMSCs infected with or without Mkx-specific shRNA.

**Results:**

Our data showed that the expression of Mkx significantly increased in hypoxic AMSCs and increased much higher in hypoxic BMSCs. Our results also detected that the expression of tenogenic differentiation markers after downregulation of Mkx were significantly decreased not only in normoxic BMSCs, but also in hypoxic BMSCs which paralleled the inferior histological evidences, worse biomechanical properties, and smaller diameters of collagen fibrils in vivo. In addition, our in vitro data demonstrated that the optical density values, the clone numbers, the percentage of cells in S phage, and cell proliferation potential of both normoxic and hypoxic BMSCs were all significantly increased after knockdown of Mkx and were also significantly enhanced in both AMSCs and BMSCs in hypoxia condition under which the expression of Mkx was upregulated.

**Conclusions:**

These findings strongly suggested that Mkx mediated hypoxia-induced tenogenic differentiation of MSCs but could not completely repress the proliferation of hypoxic MSCs.

**Supplementary Information:**

The online version contains supplementary material available at 10.1186/s13287-021-02506-3.

## Introduction

Tendon is composed of collagen fibers interspaced by little vessel [1, 2]. The forces transmission from muscles to bones in body movement and the hypovascularity make tendons tend to be subject to chronic injury, which accounts for the fact that tendon injury is a common but challenging medical problem, especially in athletes [2–4]. Tendon injury is also prevalent in the old people with the increasing incidence along the aggravation of global aging [3–5].

The self-repair of tendon injury is difficult because of the poor differentiation ability of tendon cells [1, 4, 6]. However, differentiation-inducing therapy of mesenchymal stem cells (MSCs) has been reported to be potential in treating tendon injury [2, 6, 7]. Transforming growth factor (Tgf) superfamily members, such as Tgf-β1 [8, 9], Tgf-β2 [10], Tgf-β3 [11, 12], bone-derived morphogenetic protein-12 (BMP12, i.e. growth differentiation factor-7, GDF-7) [13], BMP-13 (GDF-6) [14], and BMP-14 (GDF-5) [15] have been administrated to the injured tendon to enhance the biomechanical and histological performances. Fibroblast growth factor-2 [16, 17] and platelet-derived growth factor-BB [18] have been applied to improve tendon repair as well. However, among the various differentiation-inducing therapies, hypoxia not only has shown to be an efficient inductor in tenogenic differentiation of MSCs in repairing tendon injury [19–21], but also has been found to promote the proliferation of MSCs [19, 22–24]. The expression of tenocyte-related genes, such as tenomodulin (Tnmd) [19] and tenascin C (Tnc) [20, 21], were greater under hypoxic culture condition compared with those under normoxic culture condition. An animal study has also found that MSCs from a hypoxic culture have shown better biomechanical and histological evidences than normoxic group in Achilles tendon repair [25]. In addition, our previous study has demonstrated that, compared to Tgf-β1-treated MSCs, hypoxia-induced MSCs possessed stronger tenogenic differentiation capabilities in vitro and promoted patellar tendon repair in vivo [26]. The improved effect of hypoxia on the proliferation of MSCs has also been confirmed. Many studies have found that hypoxia increased the number of MSCs and augmented the formation of colonies [19, 22]. However, the mechanism underlying the process of hypoxia-induced tenogenesis and proliferation of MSCs is still unclear, which largely impedes the advance of differentiation-inducing therapy of MSCs in tendon injury.

Mkx gene was first found in the developing mouse embryo [27] and was widely accepted as a transcription factor in tenogenic differentiation of MSCs [1, 28, 29]. Few studies have found that Mkx was a repressor in the proliferation of MSCs. Colony-forming unit showed that Mkx-expressing MSCs significantly decreased their self-renewal capacities [30]. Many studies reported the positive correlation between the expression of Mkx and tendon-associated markers in MSCs. The tensile strength of Achilles tendon and the type I collagen (Col-1a1) productivity in Achilles tendon and tail tendon of Mkx^−/−^ mice [31] and Mkx^−/−^ rats [32] were significantly lower than those of wild-type, respectively. In addition, MSCs overexpressed Mkx displayed a significantly higher level of Col-1a1 [28], Tnmd [28], Tnc [28], and Decorin (Dcn) [32, 33]. However, whether Mkx was involved in hypoxia-induced tenogenic differentiation of MSCs has not been reported.

Hence, this study was designed to investigate the role of Mkx in hypoxia-induced tenogenic differentiation and proliferation of MSCs. Because Tgf-β1 induction was demonstrated to be a good comparison in tenogenic differentiation in our previous study [26], it was also adopted to compare with hypoxia in terms of the expression level of Mkx in this study. Our study found that hypoxia showed stronger potential than Tgf-β1 to enhance the expression of Mkx in both adipose-derived MSCs (AMSCs) and bone marrow-derived MSCs (BMSCs) and that knockdown of Mkx reduced not only the tenogenic differentiation of normoxic BMSCs but also the tenogenesis of hypoxic BMSCs in vitro and in vivo. Our results also demonstrated that Mkx remarkably repressed the proliferation of both normoxic and hypoxic BMSCs but could not completely inhibit the effect of hypoxia on proliferation of BMSCs.

## Materials and method

### Animals

Eighteen male New Zealand white rabbits were used in this study (2.5–3 kg, 3–4 months old). Before the study, all rabbits were examined for general health. AMSCs and BMSCs were collected from six rabbits. The rest rabbits were randomly allocated to either the scramble group or the short hair RNA (shRNA) group. In the following experiment, a commercial animal cage (49 cm × 35 cm × 32 cm) with free access to water and food was used for each rabbit which was housed in an animal room kept at room temperature and a 12-h:12-h light-dark cycle. This study was conducted according to the Guideline of Animal Care and Use Committee of the Fourth Military Medical University and was approved by the Ethics Committees of the Fourth Military Medical University. All efforts were made to minimize the number and the discomfort of the rabbits.

### Isolation and culture of AMSCs and BMSCs

AMSCs and BMSCs were isolated and cultured according to our previous study [26]. Briefly, AMSCs from the inguinal adipose tissue were collected and digested by type I collagenase (0.2%, Sigma, USA), filtered by 200-mesh sieve, centrifuged at 350×*g* for 5 min, resuspended in DME/F12 complete medium containing 15% fetal bovine serum (FBS, Gibco, USA) and 1% penicillin/streptomycin/amphotericin B (Cellmaxin plus, Gendepot, USA), and plated onto 10-cm cell culture dishes at 37 °C with 5% CO_2_. BMSCs were isolated from bone marrow of the femora which was flushed out with the DME/F12 complete medium. After repetitively pipetting, the bone marrow was plated onto 10-cm cell culture dishes at 37 °C with 5% CO_2_. The medium was changed every 2 days and the cells (passage 0, P0) were subcultured when 80–90% confluence was reached. The P3 cells were used in the experiment.

In hypoxic condition described in our previous study [26], MSCs were cultured in DME/F12 complete medium for 7 consecutive days in a tri-gas incubator maintained 1% O_2_, 5% CO_2_, and 94% N_2_ (MCO-5M, SANYO, Japan). Whereas in Tgf-β1 induction, MSCs were cultured in complete medium containing 10 ng/ml Tgf-β1 (Sigma, USA) for 7 consecutive days. The complete medium was changed every 2 days.

### Flow cytometry

To confirm surface marker of MSCs, flow cytometry analysis was applied according to the manufacturer’s instructions. 1 × 10^6^ cells at P3 in the logarithm growth period were collected. After washing with 1% pre-cooled FBS/PBS and centrifuging at 350×*g* for 5 min, these cells were incubated with anti-CD45-APC (Invitrogen, USA), anti-CD29-FITC (Invitrogen, USA), and anti-CD44-APC (Novus Biologicals, USA) in the dark at 4 °C for 30 min, respectively. Labeled cells were washed twice and examined using the FACScan flow cytometry system (BD, Franklin Lakes, USA). FlowJo software (TreeStar, Ashland, OR, USA) was used to analyze the data. PBS solution was used as negative control. For cell cycle analysis of DNA content, the cells were cultured for 48 h under normoxia or hypoxia condition before they were collected, washed with PBS, and resuspended with 0.3 ml PBS and 1.2 ml pre-cooled 100 % ethanol for 1 h at − 20 °C. The cells were then centrifuged (300×*g*, 5 min) and resuspended with 1 ml PBS for 15 min at room temperature. The cells were then centrifuged (300×*g*, 5 min) again. One hundred microliters RNase A (Elabscience Biotechnology Co., Ltd., China) was added to each sample which was incubated at 37 °C for 30 min. Before test, 400 μl propidium iodide (Elabscience Biotechnology Co., Ltd., China) was added to each tube at 4 °C for 30 min.

### Immunofluorescence staining

3.5-cm laser confocal dishes were used to culture and fix MSCs when cells reached 60% confluence. After blocking with Immunol Staining Blocking Buffer (containing Triton X-100 for permeabilization; Beyotime, China) for 1 h, the cells were incubated with Mkx (1:200, Aviva Systems Biology, San Diego, USA) and Tnmd (1:400, Bioss, Beijing, China) primary antibodies solution overnight at 4 °C. After washing 3 times with PBS, the cells were incubated with Cy3-goat anti-rabbit IgG (Beijing ComWin Biotech Co., Ltd. China) at room temperature for 1 h in a dark place. For counterstaining, DAPI was used for 5 min to visualize cell nuclei. The prepared samples were examined under laser scanning confocal microscope (Nikon A1R, Japan). The images (each sample for at least 3 fields) were analyzed with Image Pro Plus version 6.0 (Media Cybernetics, Inc.) as described previously [26]. The average optical density (AOD) was equal to integrated optical density over area. For CD molecular identification, the cells were blocked with normal goat serum and incubated with the following antibodies: CD45-APC (Invitrogen, USA), CD29-FITC (Invitrogen, USA), and CD90-FITC (BioLegend, USA) overnight at 4 °C in dark place.

### Multipotent differentiation

The differentiation potential of AMSCs and BMSCs towards the chondrogenic, adipogenic, and osteogenic lineages was assessed as described previously [26]. Briefly, 3 × 10^5^ MSCs were collected and centrifuged to be a pellet which was cultured in chondrogenic induction medium in the 15-mL tube for 28 days. 2 × 10^4^ cells/cm^2^ were seeded in 6-well plates, first cultured in DME/F12 complete medium and then cultured in adipogenic induction medium A for 72 h and finally cultured in adipogenic induction medium B for 24 h. The cells were cultured in turn in adipogenic induction medium A and B and were stained after repeating 5 times. For osteogenic differentiation, 2 × 10^4^ cells/cm^2^ were seeded and cultured in DME/F12 complete medium in 6-well plates which were covered by 0.1% gelatin. The culture medium was changed with osteogenic induction medium after the cells were reached 70% confluence. Alcian Blue 8GX solution, Oil Red O solution, and Alizarin Red solution were used for chondrogenic, adipogenic, and osteogenic staining, respectively.

### Cell counting kit-8 (CCK-8) assay

AMSCs and BMSCs were seeded in 96-well plates at a density of 2 × 10^3^ cells/well, respectively, and cultured in the DME/F12 complete medium at 37 °C for 7 consecutive days under normoxia condition and hypoxia condition. The culture medium was changed every 2 days. After the addition of 10 μl CCK-8 assay solution (Dojindo, Japan) in each well and the incubation for 1 h, the optical density (OD) value was measured using an Infinite M200 Pro Multifunctional microplate reader (Tecan (Shanghai) Trading Co., Ltd., China) at a wavelength of 450 nm.

### Colony formation unit assay

AMSCs and BMSCs were seeded into 6-well plates (1000 cells/well), respectively, and cultured at 37 °C under normoxia condition and hypoxia condition. In order to examine the effect of Mkx on the proliferation of BMSCs, the scramble group and the shRNA group were seeded into 6-well plates (1000 cells/well), respectively, and cultured at 37 °C under normoxia or hypoxia condition. The culture medium was changed every 3 days. After 7 days, the cells were washed twice with PBS, fixed in 4% methanol for 15 min, stained with 1% crystal violet dye (Sigma, USA) for 30 min at room temperature, and washed with PBS. The images of 6-well plates were captured to count the colonies.

### 5-Ethynyl-2′-deoxyuridine (EdU) assay

The cells were cultured for 48 h under normoxia or hypoxia condition before incubation with 50 μM EdU at 37 °C for 2 h, according to the protocol of EdU assay kit (Dalian Meilun Biotechnology Co., Ltd., China). The cells were fixed in 4% formaldehyde for 20 min and permeabilized with 0.5% TritonX-100 at room temperature for 15 min. The cells were washed with PBS, added 500 μl Click reaction cocktail to react with the EdU at room temperature for 30 min, and protected from light. Then, 500 μl 1 x Hoechest33342 was added at room temperature for 10 min and protected from light. Images of cells were obtained under a laser scanning confocal microscope (Nikon A1R, Japan).

### Quantitative real-time polymerase chain reaction (qRT-PCR)

The mRNA levels of Mkx and other tendon-specific genes were measured with qRT-PCR using the same condition as described previously [26]. Total RNA was extracted according to the manufacturer’s protocol using RNAiso plus (TaKaRa, Japan). qRT-PCR analysis was conducted using the CFX96 Real-Time PCR Detection System (Rotor-Gene Q 2plex, Germany). The primer sequences of Mkx synthesized by Sangon Biotech Co., Ltd. (Shanghai, China) were: forward: CCAGAGTGCGTGTGCTACAG; reverse: AAATGCTACCACAGGGCTGC. Other primer sequences of tendon-specific genes were same to our previous report [26]. Specificity of primers was examined by the melting curve. Data were collected from at least five independent samples and were tested at least three times. The expression levels of tendon-specific genes relative to β-actin were determined using the 2^−ΔΔCT^ method.

### Western blot

The protein expression of Mkx and other tendon-specific genes were measured with Western blot using the same condition as described previously [26]. Proteins were extracted using RIPA Lysis Buffer (Shanghai Weiao Biotechnology Co., Ltd., China). BCA protein reagent kit (Beijing Solarbio Science & Technology Co., Ltd., China) was used to measure the concentration. Thirty micrograms proteins were run on SDS-PAGE gels (8%) and transferred onto a polyvinylidene difluoride (PVDF) membrane. The PVDF membranes were blocked with 10% skim milk at room temperature for 1 h and were incubated with anti-Mkx (1:200, Aviva Systems Biology, San Diego, USA), anti- Col-1a1 (1:400, Bioss, Beijing, China), anti-Collagen type III (Col-3a1, 1:400, Bioss, Beijing, China), anti-Dcn (1:400, Bioss, Beijing, China), and anti-Tnmd (1:400, Bioss, Beijing, China) primary antibodies solution and anti-β-Actin (1:1000, BOSTER Biological Technology Co., Ltd, China) primary antibody solution overnight at 4 °C. After reacting with horseradish peroxidase-conjugated goat anti-rabbit secondary antibody or goat anti-mouse secondary antibody (BOSTER Biological Technology Co., Ltd, China) for 1 h at room temperature, proteins were detected with ECL hypersensitive chemiluminescence kit (Shanghai Weiao Biotechnology Co., Ltd., China) according to the manufacturer’s recommendations.

### RNA interference

After culturing in 6-well plates for 24 h, BMSCs were transfected with 100 nmol/L Mkx-specific small interfering RNA (siRNA, Guangzhou RiboBio Co., Ltd., China) or scrambled siRNA (negative control) using lipofectamine 3000 reagent (Invitrogen, USA) according to the manufacturer’s instruction. The siRNA sequence was 5′-GCAGCTTGTTGAACCGCTA-3′. Cells were collected 72 h after transfection and analyzed for mRNA expression by qRT-PCR and protein expression by Western blot.

### Construction of lentiviral vectors and stable infection

To stably knock down the expression of Mkx, a specific lentiviral vector (Hanbio Biotechnology Co., Ltd., Shanghai, China) containing green fluorescent protein (GFP) and stably expressed Mkx-specific shRNA was constructed with the same sequence used in siRNA. The scramble sequence was 5′-TTCTCCGAACGTGTCACGTAA-3′ (Hanbio Biotechnology Co., Ltd., Shanghai, China). BMSCs were cultured in 6-well plates for 24 h and were transduced with lentiviral particles using 8 μg/ml polybrene at a multiplicity of infection of 50 according to the manufacturer’s instructions. The expression of GFP was observed at 72 h after transduction using a fluorescence microscope (Olympus, Japan). Cells were cultured in complete medium containing 2 μg/ml puromycin (Solarbio, China) for selection.

### Tendon injury model

Bilateral patellar tendon injury model was established in the scramble group or the shRNA group following a protocol previously described [26]. Briefly, after making a longitudinal skin incision over the patellar tendon and the removal of subcutaneous fascia, the middle part of the patellar tendon was transversely severed. Fifty microliters PBS containing hypoxic BMSCs (1 × 10^6^) transduced with Mkx-scramble or Mkx-shRNA was injected into the wound gap followed by suturing the subcutaneous fascia and the skin. The rabbits were replaced to their own cages with plaster casts on bilateral legs after the operation and were given an intramuscular injection of cefazolin sodium (0.1 g/kg, q.d) for 3 consecutive days. The plaster casts were removed after 3 weeks of immobilization.

### Histological analysis

Rabbits were suffocated at 4 weeks after surgery for histological analysis. According to our previously described protocol [26], after the repaired patellar tendons were harvested, the staining of hematoxylin and eosin (H&E), Masson’s trichrome, and immunohistochemistry (IHC) for Col-1a1 and Tnmd were performed and the histological scores of H&E and IHC were evaluated. For frozen sections, the repaired patellar tendons were fixed and embedded with OCT compound and were sectioned using thermostatic freezing slicer (CryoStar NX50, Thermo).

### Biomechanical testing

Rabbits were suffocated at 4 weeks after surgery for biomechanical analysis as described in our previous study [26]. The patella-patellar tendon-tibial tubercle was harvested. After measuring the length and width of the patellar tendon, the patella and the tibial tubercle were fixed on aluminum clamps of a biomechanical testing machine (SPL-10 KN, Shimadzu, Japan). The patellar tendon was loaded until failure along the vertical axis at a displacement rate of 10 cm/min. The maximum load to failure, stiffness at failure, maximum stress, cross-sectional area, and elastic modulus were calculated.

### Transmission electron microscopy (TEM) analysis

The preparation of samples and the analysis of TEM sections were conducted following our previous protocol [26]. Briefly, after fixing, the samples were dehydrated, infiltrated, and embedded with absolute embedding medium. Three sections, obtained using ultramicrotome (Leica, Leica UC7, Germany) from each sample with a thickness of 60 μm, were stained. The cleanest section was chosen for TEM analysis (Hitachi HT7700, Japan). The software Image Pro Plus version 6.0 (Media Cybernetics, Inc) was applied to measure the fiber diameter.

### Statistical analysis

SPSS version 16.0 was applied in this study. Homogenous variances and normal distribution of data were examined using the Levene’s test and the Shapiro-Wilk test, respectively. All data were presented as the mean ± standard deviation (SD). Independent samples t-test was used and *P* < 0.05 was considered to be statistically significant.

## Results

Identification of AMSCs and BMSCs using flow cytometry, immunofluorescence staining, and commercial induction medium of tri-lineage differentiation kit was shown in Supplementary Figure 1.

### Hypoxia increased the expression level of Mkx in AMSCs and BMSCs

**Fig. 1:**
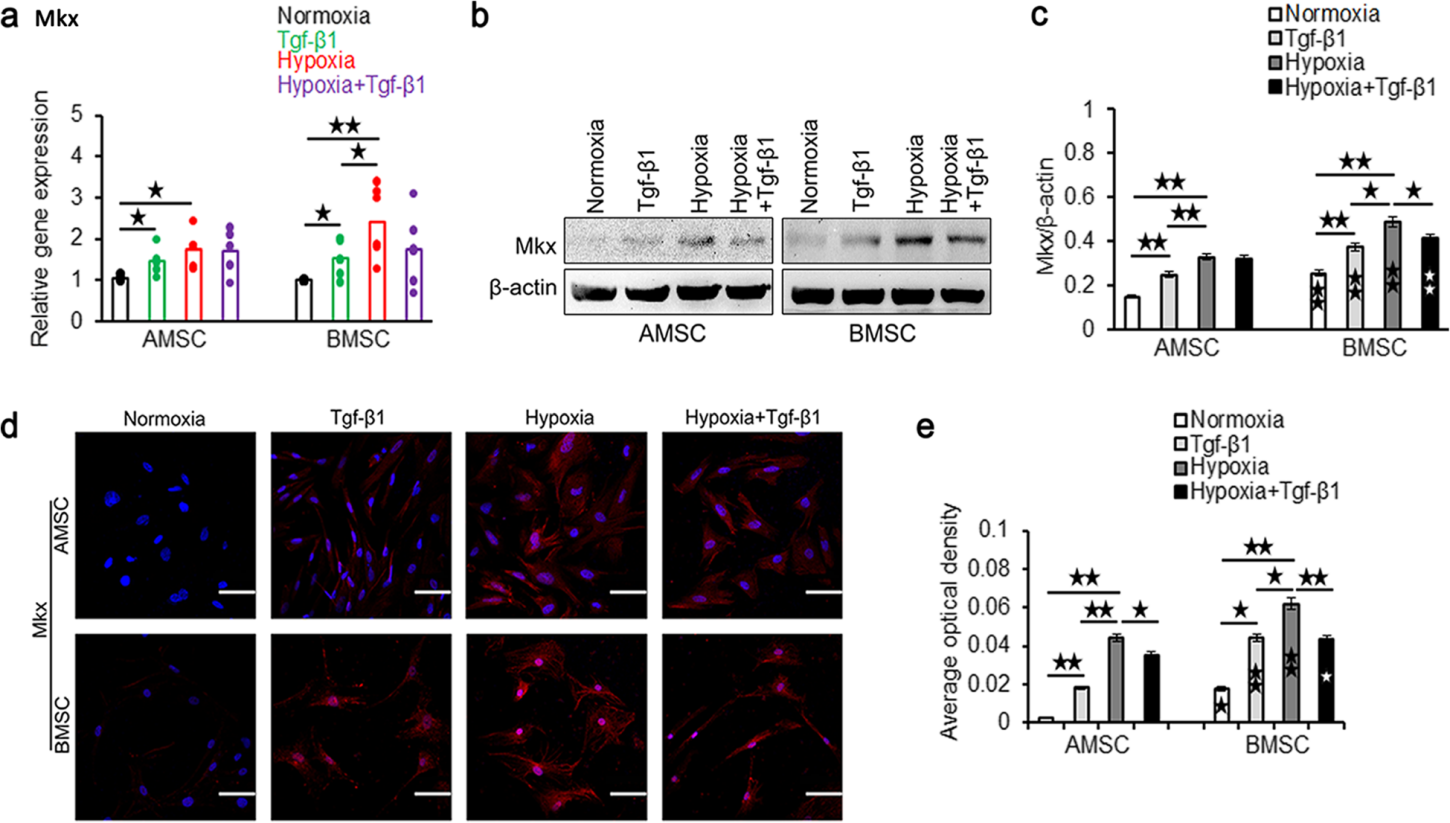
The mRNA and protein expressions of Mkx in AMSCs and BMSCs at 7 days after induction. **a** qRT-PCR analysis of gene expression of Mkx under normoxia, Tgf-β1, hypoxia, and hypoxia+Tgf-β1 condition in AMSCs and BMSCs. Representative western blots (**b**) and quantification of protein expression (**c**) of Mkx under normoxia, Tgf-β1, hypoxia, and hypoxia+Tgf-β1 condition in AMSCs and BMSCs. Asterisks in the column in BMSCs indicated significantly different from the same condition in AMSCs. Representative immunofluorescence staining (**d**) of Mkx (red) and DAPI-labeled nuclei (blue), and quantification data (**e**) under normoxia, Tgf-β1, hypoxia, and hypoxia+Tgf-β1 condition in AMSCs and BMSCs. Asterisks in the column in BMSCs indicated significantly different from the same treatment condition in AMSCs. Scale bar = 50 μm; magnification, × 400. Data were shown as mean ± SD. ^★^*p* < 0.05; ^★★^*p* < 0.01

As shown in Fig. 1a, the mRNA level of Mkx in both AMSCs and BMSCs was significantly increased in hypoxia induction than that in normoxia. Although significantly higher mRNA level of Mkx was found in Tgf-β1 induction compared to normoxia in both MSCs, the mRNA level of Mkx in Tgf-β1 induction was significantly lower than that in hypoxia induction in BMSCs. However, the mRNA level of Mkx in hypoxia and Tgf-β1 induction in both MSCs was lower than that in hypoxia induction alone but higher than that in Tgf-β1 induction alone. Similar results were found in the protein expression of Mkx in western blot analysis shown in Fig. 1b and c (Supplementary Figure 2, 3, 4, 5). Furthermore, the protein level of Mkx in all groups in BMSCs was significantly higher than that in the same group in AMSCs, respectively.

In addition, immunofluorescence staining of Mkx in both MSCs showed the similar results to western blot analysis (Fig. 1d, e). The quantitative analysis of immunofluorescence staining detected that the AOD of Mkx was significantly higher in hypoxia induction compared with the Tgf-β1 induction in AMSCs (Fig. 1e).

### Downregulation of Mkx repressed the expression level of tenogenic differentiation markers in both normoxic BMSCs and hypoxic BMSCs

**Fig. 2:**
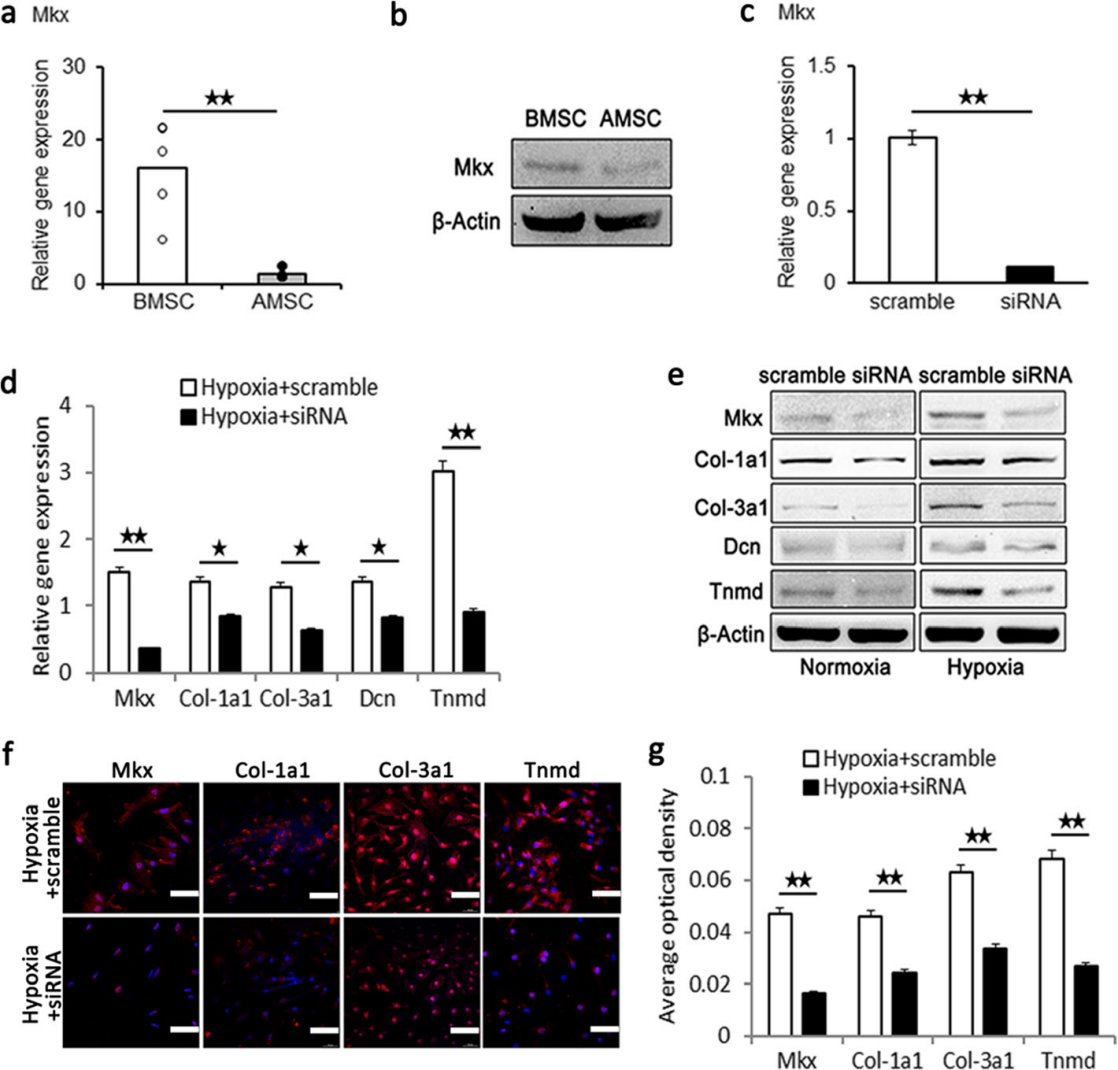
The Mkx content of AMSCs and BMSCs and the mRNA and protein expressions of Mkx and tenogenic differentiation markers after downregulation of Mkx by siRNA in BMSCs. qRT-PCR analysis (**a**) and representative western blots (**b**) of Mkx content in AMSCs and BMSCs. **c** qRT-PCR analysis of gene expression of Mkx after siRNA application in normoxia condition. **d** qRT-PCR analysis of gene expression of Mkx, Col-1a1, Col-3a1, Dcn, and Tnmd in the scramble group and the siRNA group in hypoxia condition. **e** Representative western blots of Mkx, Col-1a1, Col-3a1, Dcn, and Tnmd in the scramble group and the siRNA group in normoxia condition and in hypoxia condition. Representative immunofluorescence staining (**f**) of Mkx, Col-1a1, Col-3a1, Tnmd, and quantification data (**g**) in the scramble group and the siRNA group in hypoxia condition. Scale bar = 50 μm; magnification, × 400. Data were shown as mean ± SD. ^★^*p* < 0.05; ^★★^*p* < 0.01

As shown in Fig. 2a and b, the mRNA and protein levels of Mkx in BMSCs were significantly higher than those in AMSCs (Supplementary Figure 6). Therefore, BMSCs were selected to knock down the expression of Mkx using Mkx-specific siRNA sequences in order to investigate the role of Mkx in tenogenic differentiation in vitro.

After downregulating the mRNA levels of Mkx (Fig. 2c), the mRNA levels of Mkx and tenogenic differentiation markers, such as Col-1a1, Col-3a1, Dcn, and Tnmd were significantly lower than those in the scramble group in hypoxia induction (Fig. 2d). Similarly, the protein levels of the above tenogenic differentiation markers in the siRNA group were also significantly lower than those in the scramble group in both normoxia condition and hypoxia condition (Fig. 2e, Supplementary Figure 7, 8, 9, 10, 11, 12, 13, 14, 15, 16). As shown in Fig. 2f and g, the quantitative analysis of immunofluorescence staining detected that the AOD of Mkx, Col-1a1, Col-3a1, and Tnmd were all significantly decreased in the hypoxia and siRNA group compared with those in the hypoxia and scramble group.

### Downregulation of Mkx inhibited patellar tendon repair treated by hypoxic BMSCs

In order to study the role of Mkx in tenogenesis of hypoxic BMSCs in vivo, a specific lentiviral vector containing GFP and stably expressed Mkx-specific shRNA was constructed with the same sequence used in siRNA. The transfection efficiency was shown in Supplementary Figure 17.

#### Gross inspection and fluoroscopic inspection

**Fig. 3:**
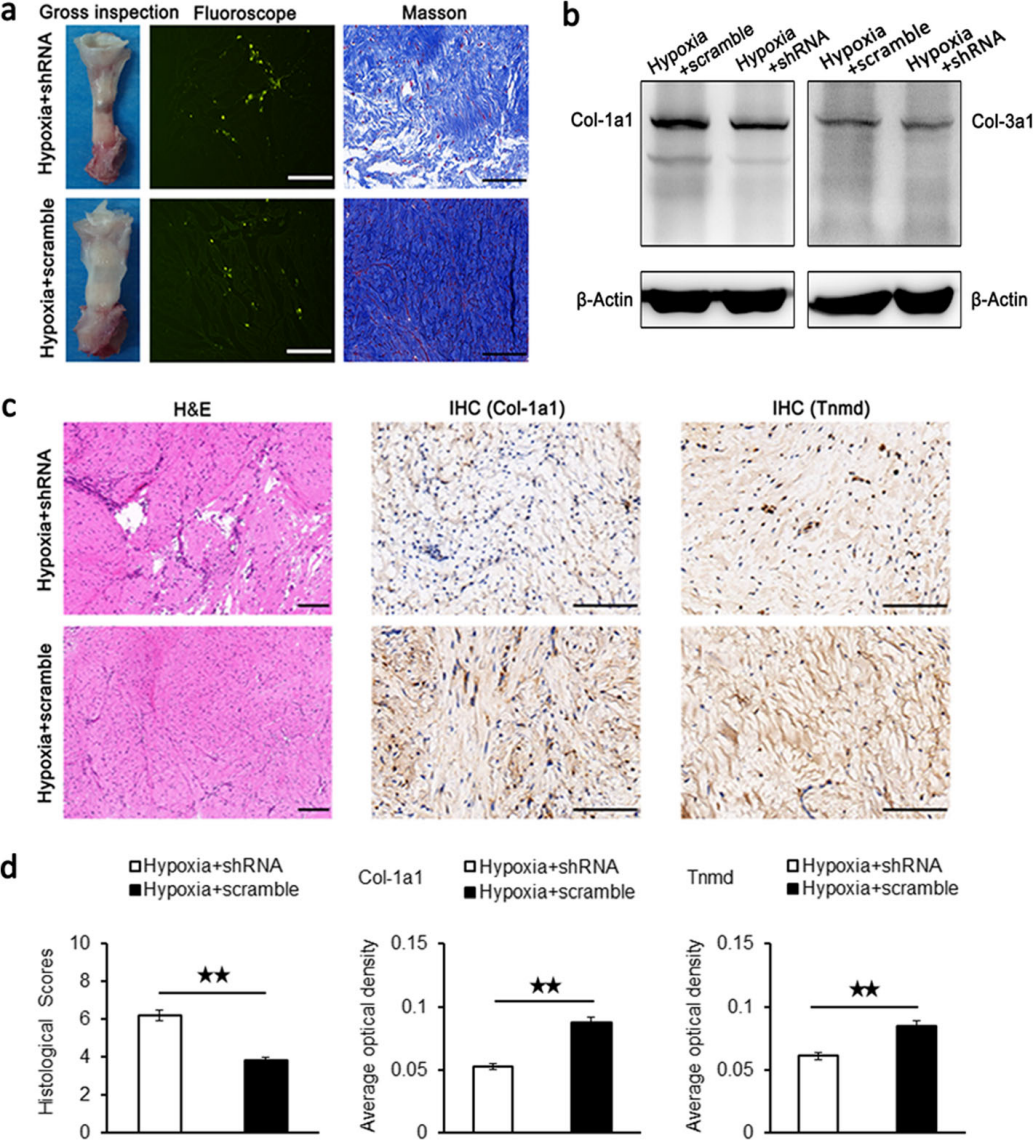
Gross observation, western blot, and histological analysis of repaired patellar tendons at 4 weeks after surgery. **a** Gross observation, frozen sections observed by fluorescence microscopy, and Masson’s staining of the hypoxia and scramble group and the hypoxia and shRNA group. Scale bar = 200 μm; magnification, × 400. **b** Representative western blots of Col-1a1 and Col-3a1 of the hypoxia and scramble group and the hypoxia and shRNA group. **c** H&E staining and IHC staining for Col-1a1 and Tnmd of the hypoxia and scramble group and the hypoxia and shRNA group. H&E staining: scale bar = 200 μm; magnification, × 200; IHC staining: scale bar = 200 μm; magnification, ×400. **d** Histological scores of H&E staining and quantification of Col-1a1 and Tnmd after IHC staining in the hypoxia and scramble group and the hypoxia and shRNA group. Data were shown as mean ± SD. ^★★^*p* < 0.01

As shown in Fig. 3a, there was a remarkable defect in the patellar tendon at 4 weeks after surgery when seeded hypoxic BMSCs with Mkx-specific shRNA infection in the wound of patellar tendon. However, when applied hypoxic BMSCs with scramble infection into wound gap, the injured patellar tendon recovered better under gross observation. The frozen sections of the hypoxia and shRNA group and the hypoxia and scramble group were observed under fluorescence microscopy, and the results showed that fluorescence could be seen at 4 weeks after surgery.

#### Masson staining and H&E staining

The histological difference between the hypoxia and shRNA group and the hypoxia and scramble group was evaluated by Masson staining and H&E staining. To detect the formation of tendon-like tissues, Masson staining was adopted. As shown in Fig. 3a, less fibrous matrix stained in blue and more empty spaces were found in the hypoxia and shRNA group, compared to those in the hypoxia and scramble group. Western blots results showed that the protein level of Col-1a1 and Col-3a1 in the hypoxia and scramble group was higher than that in the hypoxia and shRNA group, respectively (Fig. 3b). In H&E staining (Fig. 3c), similar large empty spaces and relatively fewer number of tendon cells were detected in the hypoxia and shRNA group with disorder collagen fibers and bold vessels. However, in the hypoxia and scramble group, the collagen fibers were arranged more regularly, within which less empty spaces and more cells were observed. In the following quantitative analysis (Fig. 3d), the histological scores of the hypoxia and shRNA group were significantly higher than that of the hypoxia and scramble group.

#### IHC staining

In order to determine the difference of tenogenic differentiation, IHC staining of tendon-specific markers Col-1a1 and Tnmd was performed (Fig. 3c). The staining of Col-1a1 and Tnmd was lighter in the hypoxia and shRNA group and became deeper in the hypoxia and scramble group. The average optical density of Col-1a1 and Tnmd in the hypoxia and shRNA group was significantly lower than that in the hypoxia and scramble group, respectively (Fig. 3d).

#### Biomechanical properties and ultrastructural morphology of repaired patellar tendons

**Fig. 4:**
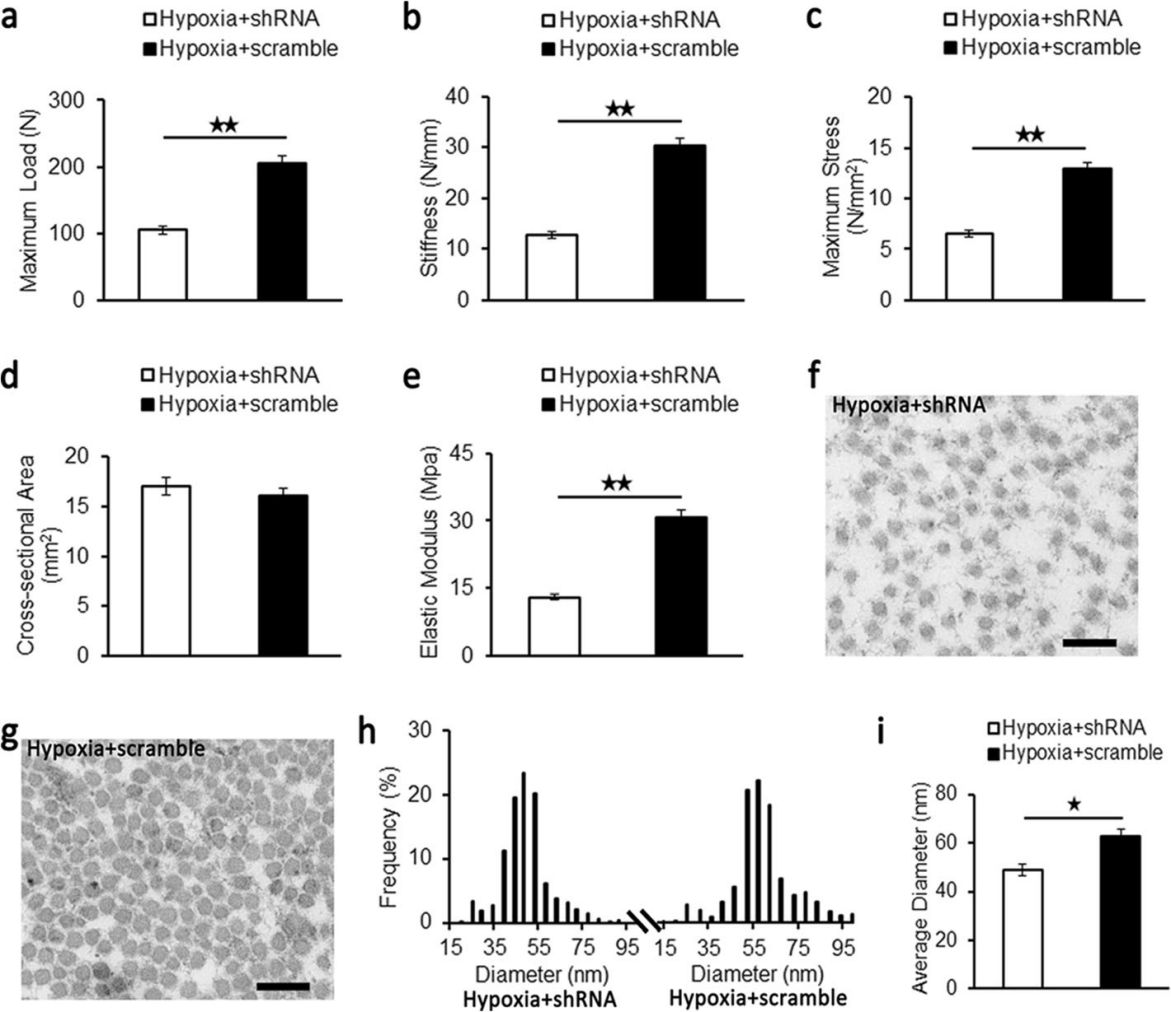
Biomechanical analysis and ultrastructure of repaired patellar tendons at 4 weeks after surgery. Biomechanical analysis for maximum load (**a**), stiffness (**b**), maximum stress (**c**), cross-sectional area (**d**), and elastic modulus (**e**) in the hypoxia and scramble group and the hypoxia and shRNA group. Representative images of transmission electron microscopy of the hypoxia and shRNA group (**f**) and the hypoxia and scramble group (**g**). Scale bar = 200 nm; magnification, × 15,000. **h** The distribution of collagen fibril diameters of the hypoxia and shRNA group and the hypoxia and scramble group. **i** The average diameter of collagen fibrils of the hypoxia and shRNA group and the hypoxia and scramble group. Data were shown as mean ± SD. ^★^*p* < 0.05; ^★★^*p* < 0.01

The maximum load (Fig. 4a), stiffness (Fig. 4b), maximum stress (Fig. 4c), and elastic modulus (Fig. 4e) were significantly lower in the hypoxia and shRNA group than those in the hypoxia and scramble group. Although no significant difference was found, the cross-sectional area was higher in the hypoxia and shRNA group than that in the hypoxia and scramble group (Fig. 4d). The diameters of collagen fibrils in repaired patellar tendons at 4 weeks after surgery were observed under transmission electron microscopy, as shown in Fig. 4f–i. After calculating the range of fibrils diameters, the results revealed that the majority of fibrils diameters ranged from 39 to 58 nm in the hypoxia and shRNA group (Fig. 4f, h) and 46 to 78 nm in the hypoxia and scramble group (Fig. 4g, h). As shown in Fig. 4i, the average diameter of collagen fibrils in the hypoxia and shRNA group was significantly smaller than that in the hypoxia and scramble group.

### Hypoxia promoted the proliferation of AMSCs and BMSCs

**Fig. 5:**
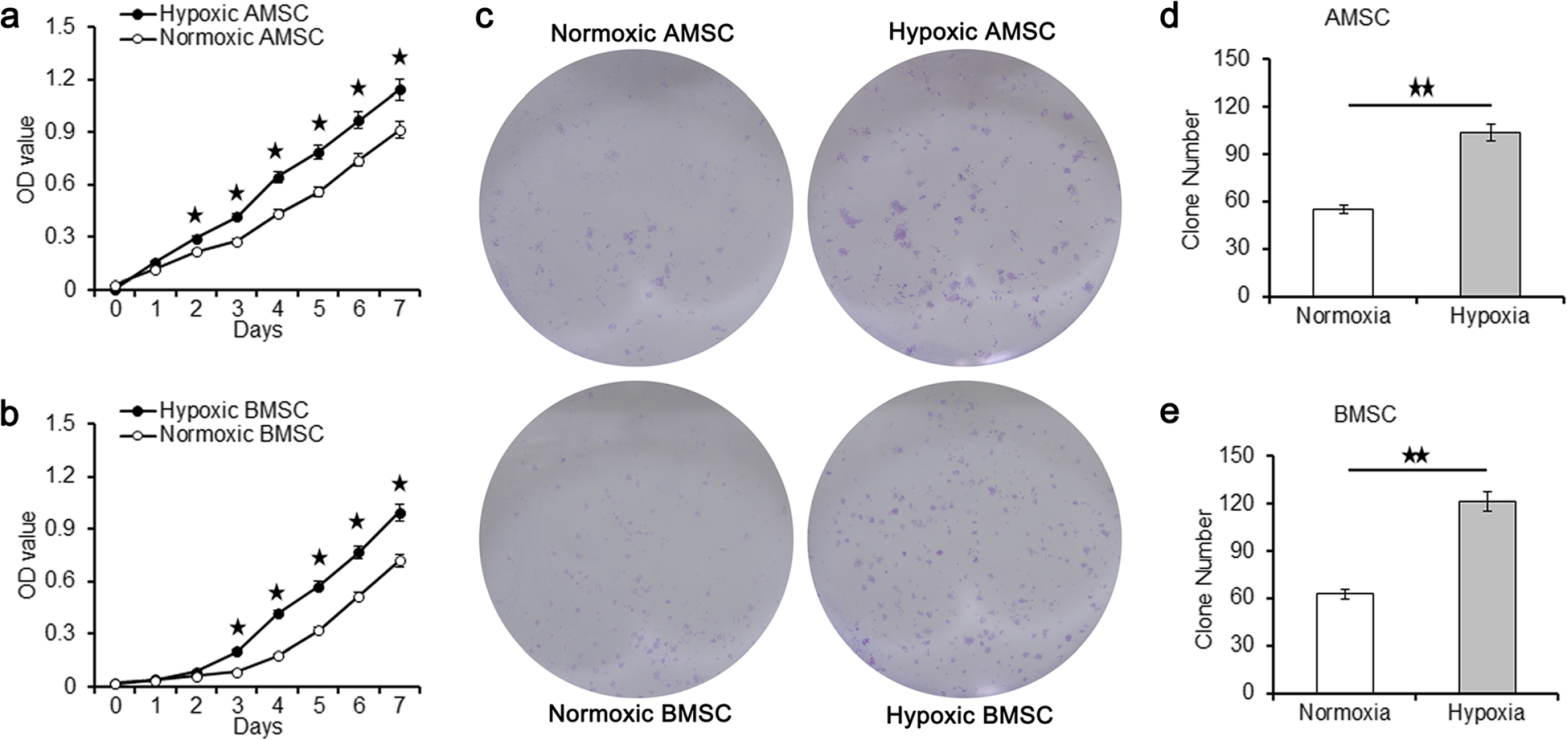
The OD values and the clone numbers of AMSCs and BMSCs under normoxia condition and hypoxia condition. The OD values of AMSCs (**a**) and BMSCs (**b**) under normoxia condition and hypoxia condition. **c** Clone formation of AMSCs and BMSCs under normoxia condition and hypoxia condition. Clone numbers of AMSCs (**d**) and BMSCs (**e**) under normoxia condition and hypoxia condition. Data were shown as mean ± SD. ^★^*p* < 0.05; ^★★^*p* < 0.01

At the second day, the OD value of hypoxic AMSCs was significantly higher than that of normoxic AMSCs according to the CCK-8 assay shown in Fig. 5a. The significant increase in OD value of hypoxic AMSCs lasted 5 days until the end of CCK-8 test. Similarly, the OD value of hypoxic BMSCs was significantly higher than that of normoxic BMSCs from the third day to the seventh day (Fig. 5b). After 7 consecutive days of hypoxic culture, the number of colony formation was significantly higher than that of normoxic culture in both AMSCs and BMSCs, as shown in Fig. 5c–e.

**Fig. 6:**
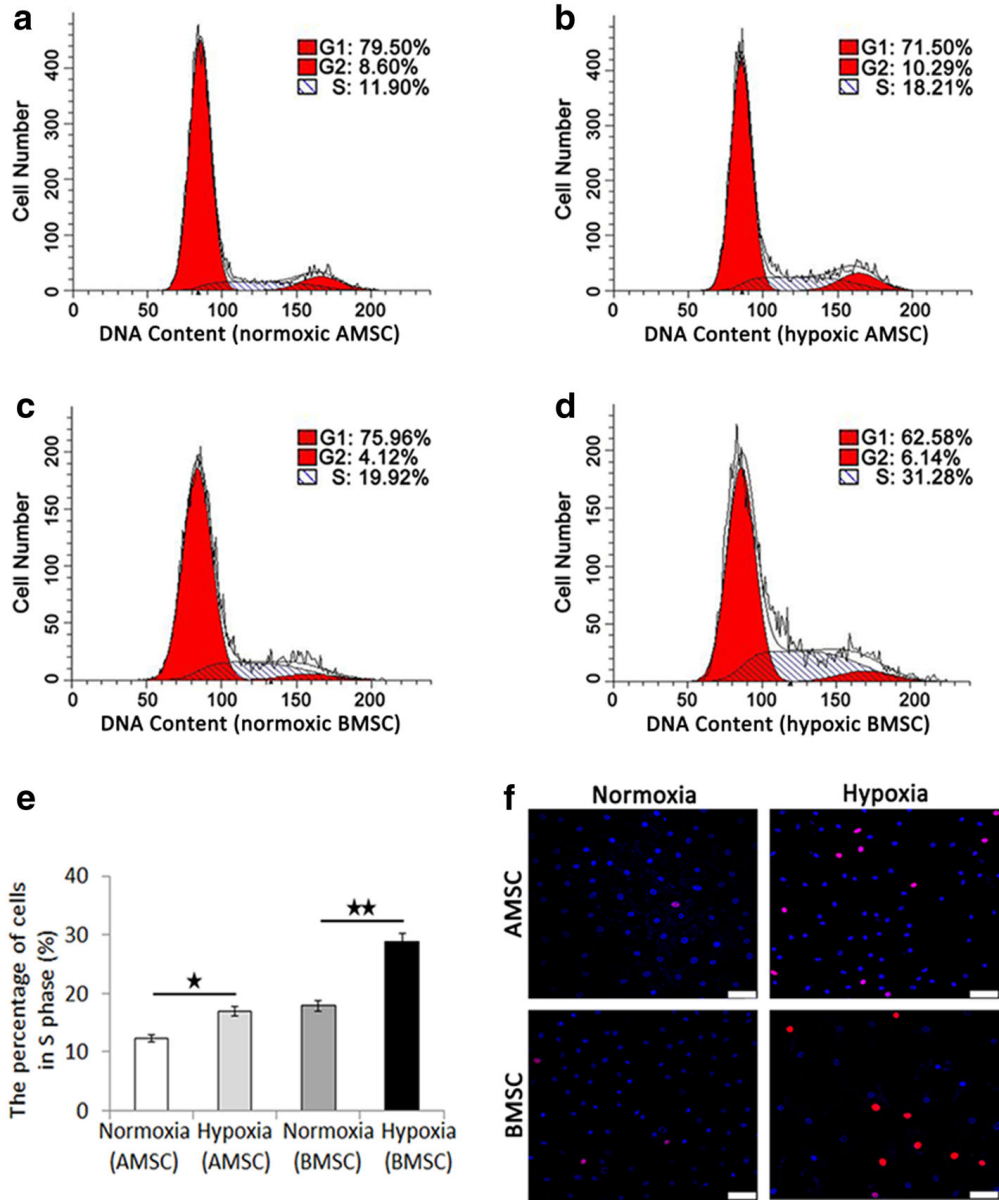
Flow Cytometry analysis of the cell cycle by DNA content and EdU assay of AMSCs and BMSCs under normoxia condition and hypoxia condition. The percentage of cells in S phage of AMSCs under normoxia condition (**a**) and hypoxia condition (**b**), BMSCs under normoxia condition (**c**) and hypoxia condition (**d**), and the quantification data of the percentage of cells in S phage (**e**). **f** EdU assay of AMSCs and BMSCs under normoxia condition and hypoxia condition. Scale bar = 50 μm; magnification, × 400. Data were shown as mean ± SD. ^★^*p* < 0.05; ^★★^*p* < 0.01

The percentage of cells in S phage of AMSCs under normoxia condition (Fig. 6a) and hypoxia condition (Fig. 6b), BMSCs under normoxia condition (Fig. 6c), and hypoxia condition (Fig. 6d) were examined using flow cytometry analysis of the cell cycle by DNA content. The quantification data showed that the percentage of cells in S phage under hypoxia condition was significantly higher than that under normoxia condition in both AMSCs and BMSCs (Fig. 6e). EdU assay detected that hypoxic AMSCs and hypoxic BMSCs possessed higher proliferation potential than that of normoxic AMSCs and normoxic BMSCs, respectively (Fig. 6f).

### Downregulation of Mkx promoted the proliferation of BMSCs in normoxia and hypoxia condition

**Fig. 7:**
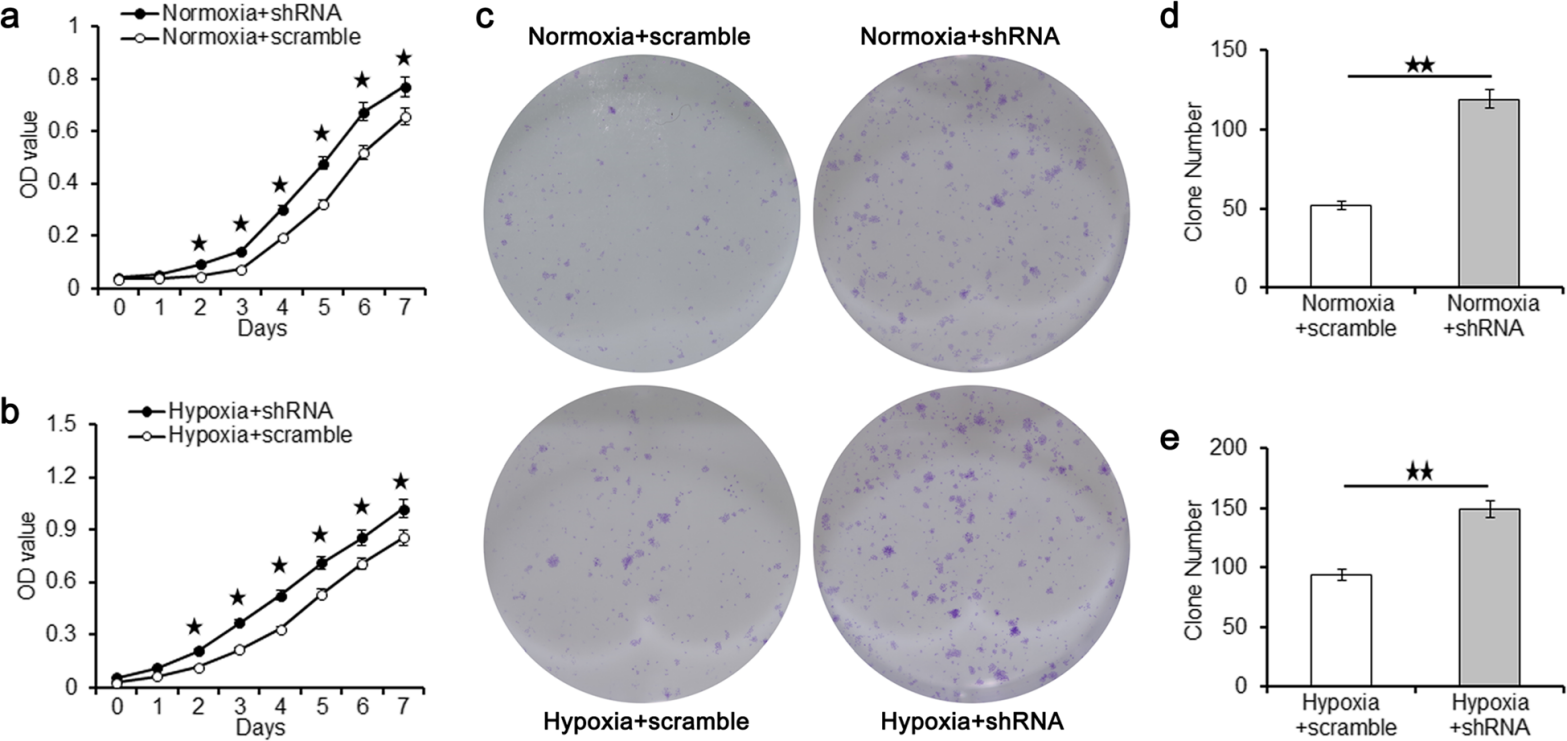
The OD values and the clone numbers of the scramble group and the shRNA group under normoxia condition and hypoxia condition. The OD values of the scramble group and the shRNA group under normoxia condition (**a**) and hypoxia condition (**b**). **c** Clone formation of the scramble group and the shRNA group under normoxia condition and hypoxia condition. Clone numbers of the scramble group and the shRNA group under normoxia condition (**d**) and hypoxia condition (**e**). Data were shown as mean ± SD. ^★^*p* < 0.05; ^★★^*p* < 0.01

As shown in Fig. 7a, the OD value of shRNA group was significantly higher than that of the scramble group at the second day according to the CCK-8 assay in normoxia condition. The significant increase in OD value of the shRNA group lasted 5 days until the end of CCK-8 test. Similarly, the OD value of shRNA group was significantly higher than that of scramble group from the second day to the seventh day in hypoxia condition (Fig. 7b). After culturing for 7 consecutive days, the number of colony formation in shRNA group was significantly higher than that in scramble group in both normoxia and hypoxia condition, as shown in Fig. 7c–e.

**Fig. 8:**
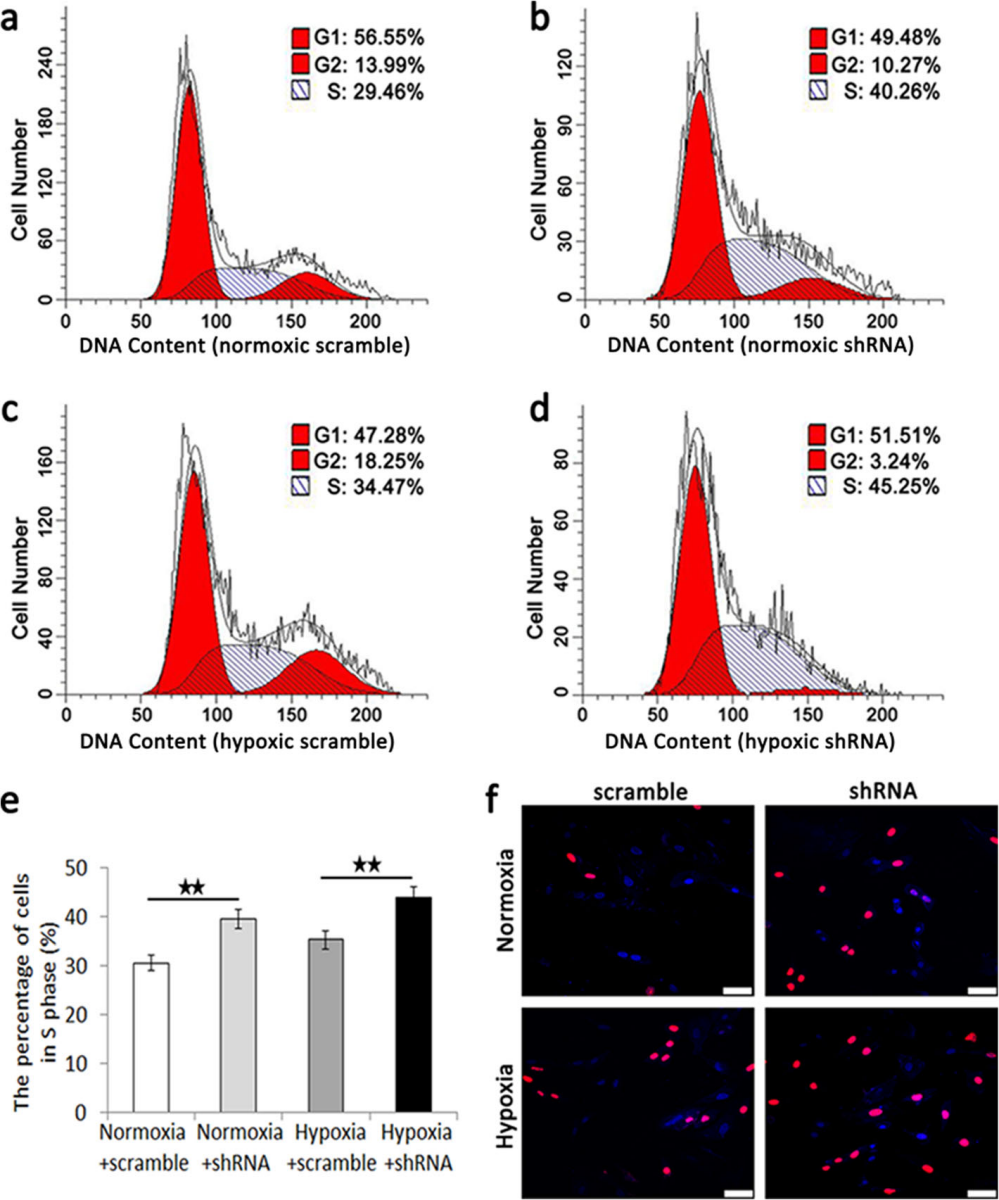
Flow Cytometry analysis of the cell cycle by DNA content and EdU assay of the scramble group and the shRNA group under normoxia condition and hypoxia condition. The percentage of cells in S phage of the scramble group (**a**) and the shRNA group (**b**) under normoxia condition and the scramble group (**c**) and the shRNA group (**d**) under hypoxia condition and the quantification data of the percentage of cells in S phage (**e**). **f** EdU assay of the scramble group and the shRNA group under normoxia condition and hypoxia condition. Scale bar = 50 μm; magnification, × 400. Data were shown as mean ± SD. ^★★^*p* < 0.01

The percentage of cells in S phage of the scramble group (Fig. 8a) and the shRNA group (Fig. 8b) under normoxia condition and the scramble group (Fig. 8c) and the shRNA group (Fig. 8d) under hypoxia condition were examined using flow cytometry analysis of the cell cycle by DNA content. The quantification data showed that the percentage of cells in S phage of the shRNA group was significantly higher than that of the scramble group in both normoxia and hypoxia condition (Fig. 8e). EdU assay detected that the shRNA group possessed higher proliferation potential than that of the scramble group in both normoxia and hypoxia condition (Fig. 8f).

## Discussion

Although the improved effects of hypoxia on tenogenic differentiation of MSCs have been reported [19–21, 26], none of them conducted research about the underlying mechanism of tenogenesis in hypoxia condition. In the present study, we detected that the expression level of Mkx was significantly enhanced in hypoxic AMSCs and hypoxic BMSCs in comparison to that of normoxic MSCs, respectively, and that the hypoxia and shRNA group showed significantly lower expression of tendon-specific markers in vitro and worse histological and biomechanical properties in vivo than those of the hypoxia and scramble group in BMSCs. Our in vitro results further showed that the OD values, the clone numbers, the percentage of cells in S phage, and cell proliferation potential of BMSCs were all significantly increased after downregulation of Mkx in both normoxia and hypoxia condition and were also significantly enhanced in AMSCs and BMSCs in hypoxia condition under which the expression of Mkx was promoted.

Many studies have proved that transcription factors Scleraxis (Scx) played an important role in promoting tenogenic differentiation of MSCs [30, 34]. However, Mkx has been shown to be more effective than Scx in inducing tenogenic differentiation of MSCs and could dramatically enhance the expression level of Scx [30]. In our study, we first found that the Mkx content of BMSCs was significantly higher than that of AMSCs, and that the protein expression of Mkx in hypoxic BMSCs was also significantly higher than that in hypoxic AMSCs. Our results further showed that the expression of tendon-specific markers, such as Col-1a1, Col-3a1, Dcn, and Tnmd were all decreased in normoxic BMSCs when Mkx was downregulated. This was similar to previous results which showed that decreased type I collagen productivity was found in Mkx mutant mice [31] and Mkx mutant rats [32]. Similarly, we detected that the expression levels of the above tendon-specific markers were also reduced in the hypoxia and shRNA group in BMSCs, compared with the hypoxia and scramble group. This indicated that Mkx was crucial for hypoxia-induced tenogenesis of BMSCs.

As a comparative inducer of tenogenesis used in our previous study [26], Tgf-β1 was found to promote the expression of Mkx in our study as well. As one of the Tgf-β superfamily members, Tgf-β1 shares similar biological structures with Tgf-β2 and Tgf-β3 isoforms [35]. This indicated that they may have the similar inducing effect on the tenogenic differentiation of MSCs. Moreover, we studied the effects of BMP-12, another Tgf-β superfamily member, on the tenogenic differentiation of MSCs in terms of mRNA level. We found that the mRNA expression levels of Col-1a1, Col-3a1, and Mkx were significantly increased in the BMP-12 group, compared with the control group (Supplementary Figure 18). Considering the proved fact that Mkx was crucial for hypoxia-induced tenogenesis of BMSCs, these results suggested that the upregulated expression of Mkx may be a reason of Tgf-β superfamily members for their improved effect on tenogenic differentiation of MSCs.

In order to better examine the formation of tendon-like tissues directly from tenogenesis of the implanted BMSCs in our study, the GFP-labeled hypoxia-induced BMSCs with Mkx-specific shRNA or scramble were seeded in the wound gap of patellar tendons. At 4 weeks after the surgery, we found that the green GFP fluorescence could still be seen in the frozen sections of the hypoxia and shRNA group and the hypoxia and scramble group under fluorescence microscopy. This paralleled a previous finding which revealed that GFP-labeled MSCs can be observed at 4 weeks after transplantation [36]. In addition, the worse histological evidences and the lower scores of Col-1a1 and Tnmd after IHC staining in the hypoxia and shRNA group corresponded to the following inferior biomechanical properties and the smaller fibril diameters. Although the biomechanical properties of the hypoxia and scramble group, which exhibited better histological findings than the hypoxia and shRNA group, were significantly lower than those of the normal control group (Supplementary Table 1), they were significantly higher than those of the hypoxia and shRNA group. This indicated that our in vivo findings validated the role of Mkx in hypoxia-induced tenogenic differentiation of BMSCs in vitro.

Tnmd was considered to be an important and specific collagen matrix in tendon formation [19, 26]. In our previous study, Tnmd was found to be the highest expression gene of MSCs in mRNA and protein level under hypoxia condition [26]. In the present study, we also examined the expression of Tnmd after the downregulation of Mkx under hypoxia condition. The results showed that it was the gene that changed the most in mRNA and protein levels before and after the downregulation of Mkx under hypoxia condition and almost no red fluorescence (Tnmd) was seen in the hypoxia and shRNA group in BMSCs after immunofluorescence staining, compared with the hypoxia and scramble group. In addition, the mRNA expression level of Tnmd was found no significant difference between the BMP-12 group and the control group (Supplementary Figure 18). These results demonstrated that hypoxia improved tenogenic differentiation of MSCs, which was mediated by Mkx, mainly by promoting the expression of Tnmd.

Many studies have reported that hypoxia prompted the self-renewal of MSCs [19, 22–24]. This was consistent with our findings which showed that the significantly higher OD values, clone numbers, percentage of cells in S phage, and cell proliferation potential were detected in both AMSCs and BMSCs in hypoxia condition, compared with those in normoxia condition. Previous study reported that Mkx has been shown to be able to inhibit the colony formation numbers of MSCs [30]. This finding was identical with our results which showed that the OD values, the clone numbers, the percentage of cells in S phage and cell proliferation potential of the shRNA group were all significantly higher than those of the scramble group in both normoxia and hypoxia condition. However, the remarkably increased level of Mkx and the significantly higher OD values, clone numbers, percentage of cells in S phage, and cell proliferation potential of the hypoxic BMSCs, compared with those of the normoxic BMSCs in our study, indicated that Mkx could not completely inhibit the effect of hypoxia on proliferation.

## Conclusion

In conclusion, we have investigated the role of Mkx in hypoxia-induced tenogenic differentiation and proliferation of MSCs. Our data showed that the expression of Mkx significantly enhanced in AMSCs and BMSCs under hypoxia condition in vitro and that Mkx mediated hypoxia-induced tenogenic differentiation of BMSCs in vitro and in vivo but incompletely inhibited the proliferation of hypoxic BMSCs in vitro.

## Supplementary Information

**Additional file 1: Supplementary Figure 1**: Identification of AMSCs and BMSCs.

**Additional file 2: Supplementary Figure 2**: Western blot of β-Actin in AMSCs.

**Additional file 3: Supplementary Figure 3**: Western blot of Mkx in AMSCs.

**Additional file 4: Supplementary Figure 4**: Western blots of β-Actin in BMSCs.

**Additional file 5: Supplementary Figure 5**: Western blots of Mkx in BMSCs.

**Additional file 6: Supplementary Figure 6**: Western blot of Mkx content in AMSCs and BMSCs.

**Additional file 7: Supplementary Figure 7**: Western blot of Mkx under normoxia condition.

**Additional file 8: Supplementary Figure 8**: Western blot of Col-1a1 under normoxia condition.

**Additional file 9: Supplementary Figure 9**: Western blots of Col-3a1 and β-Actin under normoxia condition.

**Additional file 10: Supplementary Figure 10**: Western blot of Dcn under normoxia condition.

**Additional file 11: Supplementary Figure 11**: Western blot of Tnmd under normoxia condition.

**Additional file 12: Supplementary Figure 12**: Western blot of Mkx under hypoxia condition.

**Additional file 13: Supplementary Figure 13**: Western blot of Col-1a1 under hypoxia condition.

**Additional file 14: Supplementary Figure 14**: Western blot of Col-3a1 under hypoxia condition.

**Additional file 15: Supplementary Figure 15**: Western blots of Dcn and Tnmd under hypoxia condition.

**Additional file 16: Supplementary Figure 16**: Western blots of β-Actin under hypoxia condition.

**Additional file 17: Supplementary Figure 17**: The transfection efficiency of Mkx-specific shRNA in BMSCs.

**Additional file 18: Supplementary Figure 18**: Gene expression of Mkx and tenogenic differentiation markers under BMP-12 condition in BMSCs.

**Additional file 19: Supplementary Table 1**: Comparison of the biomechanical properties of the hypoxia and shRNA group, the hypoxia and scramble group and the normal control groups in patellar tendon.

## Data Availability

The datasets generated during the current study are available from the corresponding author on reasonable request.

## Abbreviations

AMSCs: Adipose-derived MSCs
AOD: Average optical density
BMP-12: Bone-derived morphogenetic protein-12
BMSCs: Bone marrow-derived MSCs
CCK-8: Cell counting kit-8
Col-1a1: Collagen type I
Col-3a1: Collagen type III
Dcn: Decorin
EdU: 5-Ethynyl-2′-deoxyuridine
FBS: Fetal bovine serum
GDF: Growth differentiation factor
GFP: Green fluorescent protein
H&E: Hematoxylin and eosin
IHC: Immunohistochemistry
Mkx: Mohawk homeobox
MSCs: Mesenchymal stem cells
OD: Optical density
P: Passage
PBS: Phosphate buffer saline
PVDF: Polyvinylidene difluoride
qRT-PCR: Quantitative real-time polymerase chain reaction
Scx: Scleraxis
SD: Standard deviation
shRNA: Short hair RNA
siRNA: Small interfering RNA
TEM: Transmission electron microscopy
Tgf-β: Transforming growth factor-β
Tnc: Tenascin C
Tnmd: Tenomodulin
