# Supplementary figures and images for: Mkx mediates tenogenic differentiation but incompletely inhibits the proliferation of hypoxic MSCs
Source: Stem Cell Res Ther. 2021 Jul 28;12:426. doi: 10.1186/s13287-021-02506-3 (PMC8317301; doi:10.1186/s13287-021-02506-3)

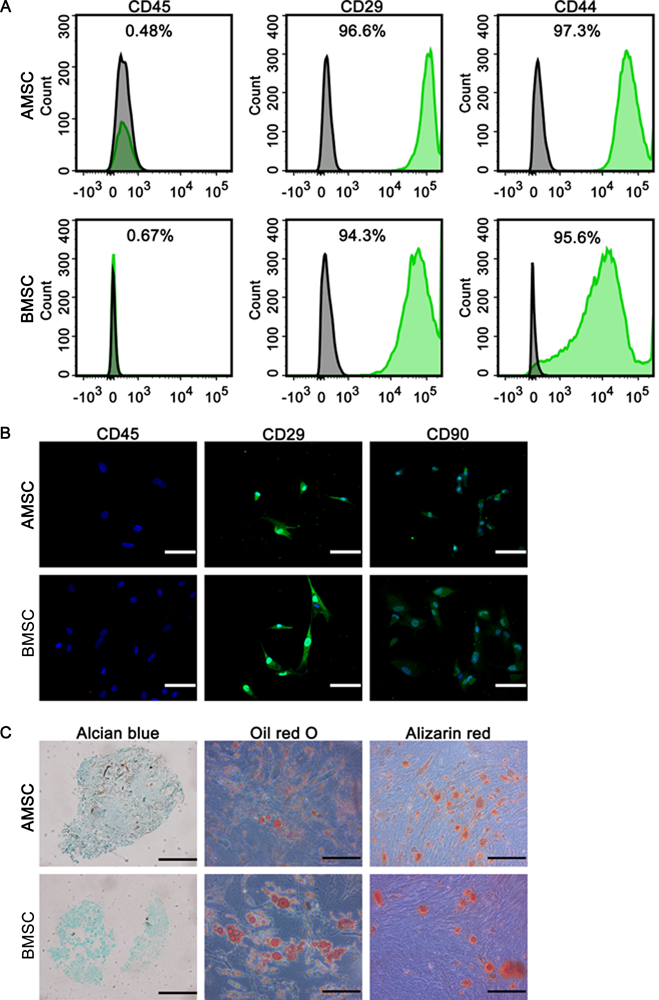

Supplement: Supplementary file 1 — Additional file 1: Supplementary Figure 1: Identification of AMSCs and BMSCs. [file 13287_2021_2506_MOESM1_ESM.tif]

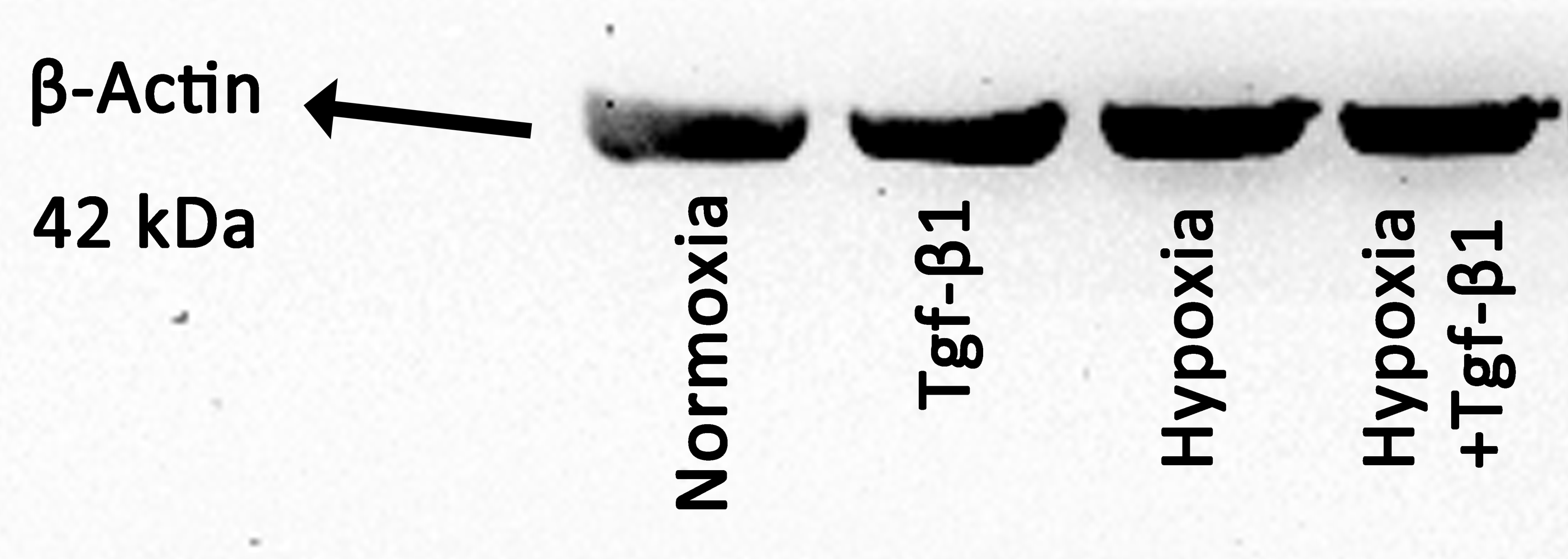

Supplement: Supplementary file 2 — Additional file 2: Supplementary Figure 2: Western blot of β-Actin in AMSCs. [file 13287_2021_2506_MOESM2_ESM.tif]

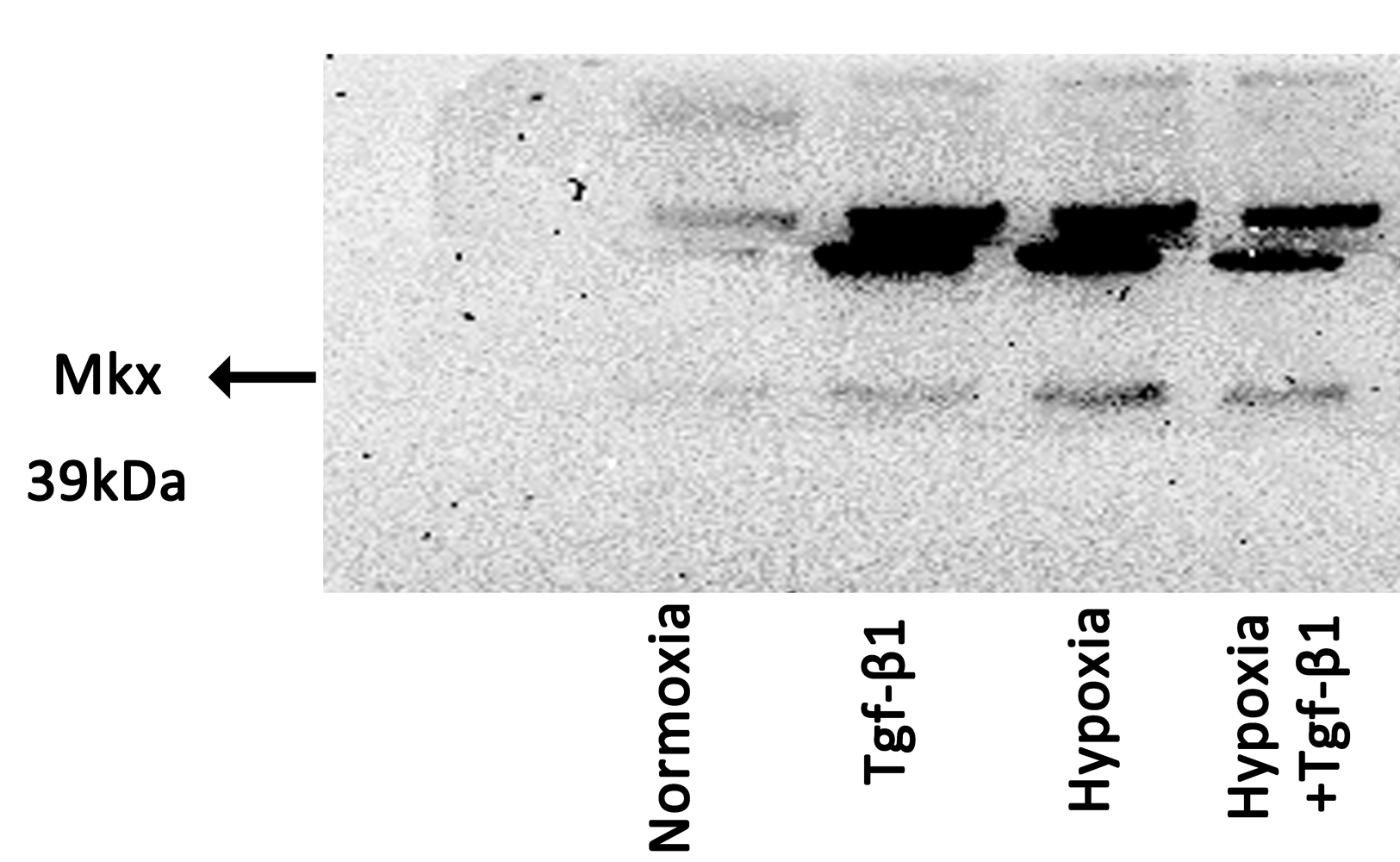

Supplement: Supplementary file 3 — Additional file 3: Supplementary Figure 3: Western blot of Mkx in AMSCs. [file 13287_2021_2506_MOESM3_ESM.tif]

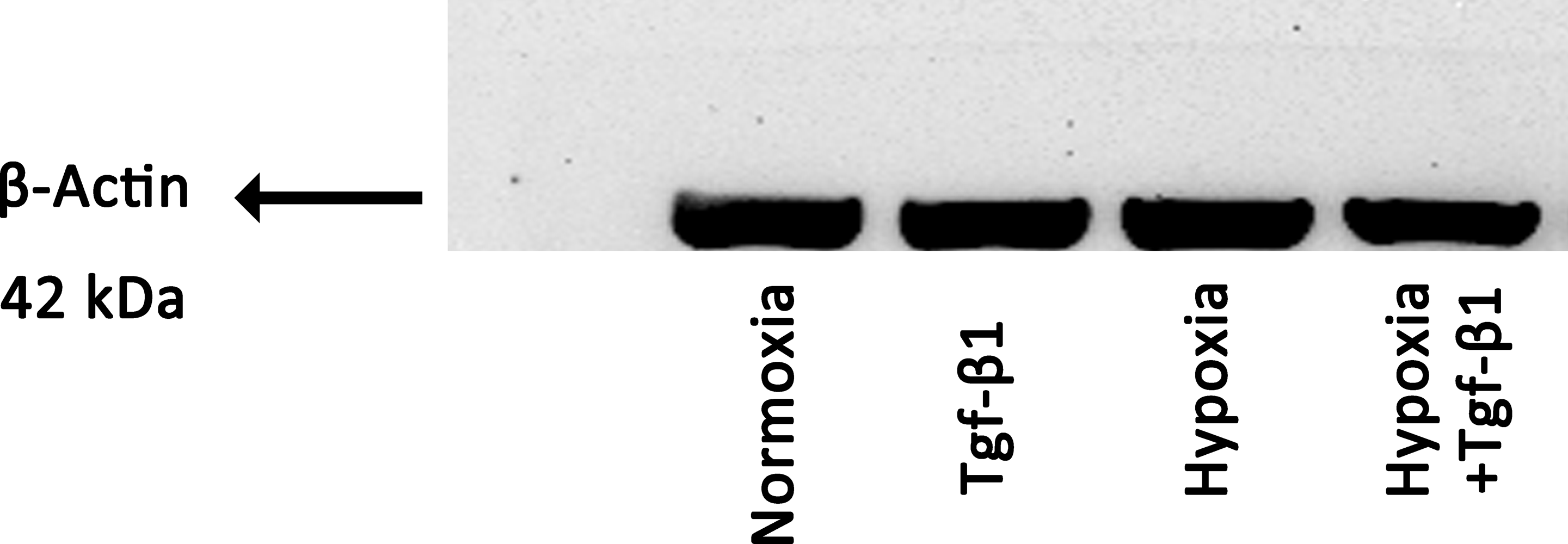

Supplement: Supplementary file 4 — Additional file 4: Supplementary Figure 4: Western blots of β-Actin in BMSCs. [file 13287_2021_2506_MOESM4_ESM.tif]

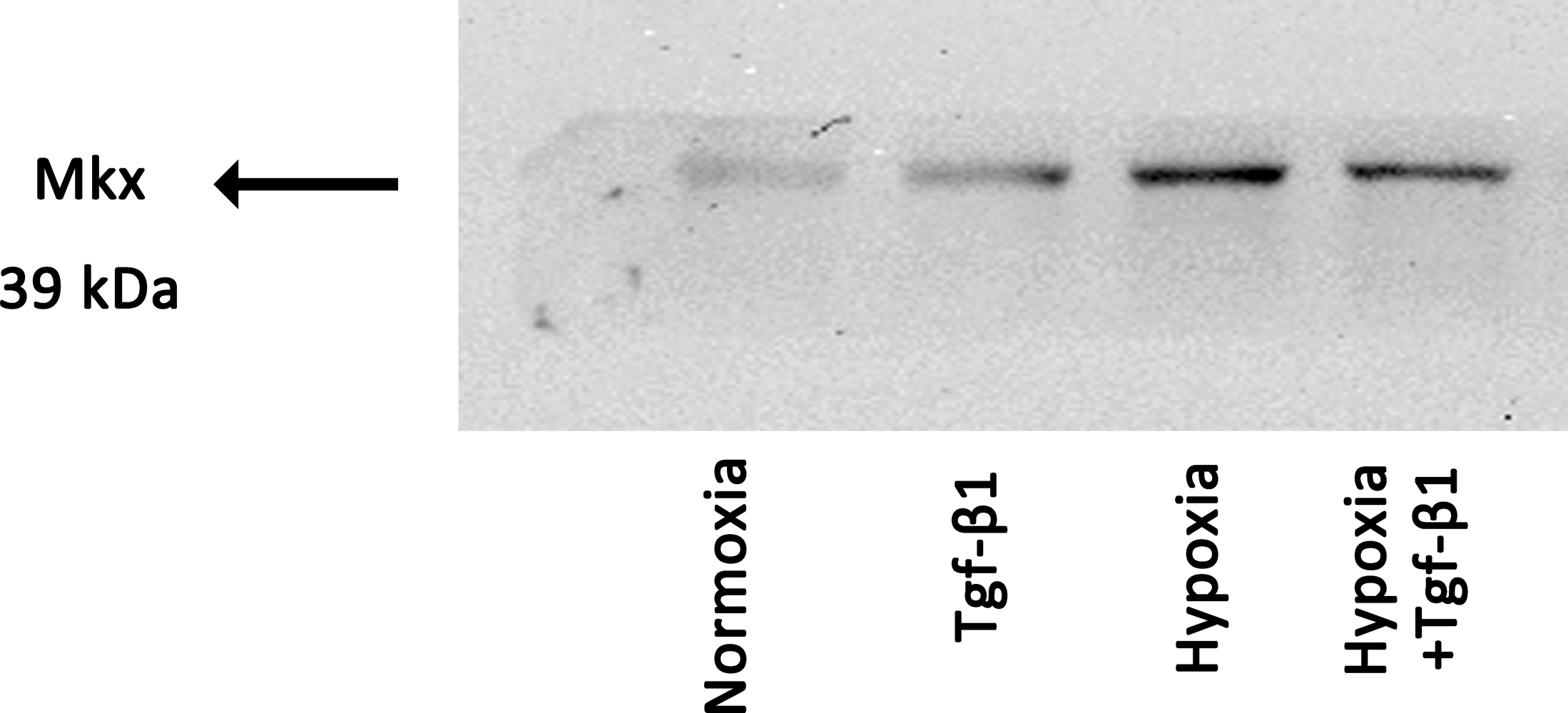

Supplement: Supplementary file 5 — Additional file 5: Supplementary Figure 5: Western blots of Mkx in BMSCs. [file 13287_2021_2506_MOESM5_ESM.tif]

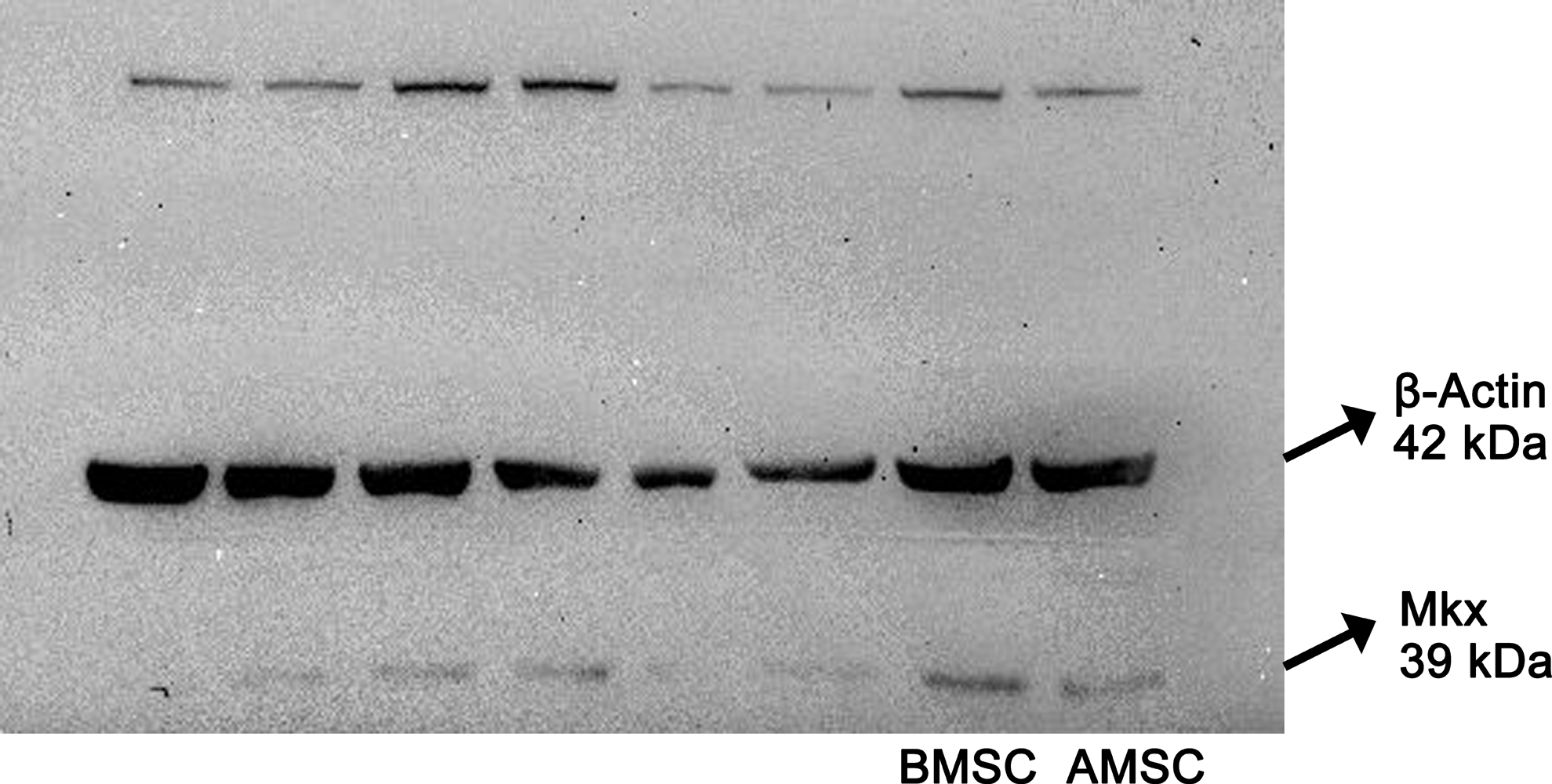

Supplement: Supplementary file 6 — Additional file 6: Supplementary Figure 6: Western blot of Mkx content in AMSCs and BMSCs. [file 13287_2021_2506_MOESM6_ESM.tif]

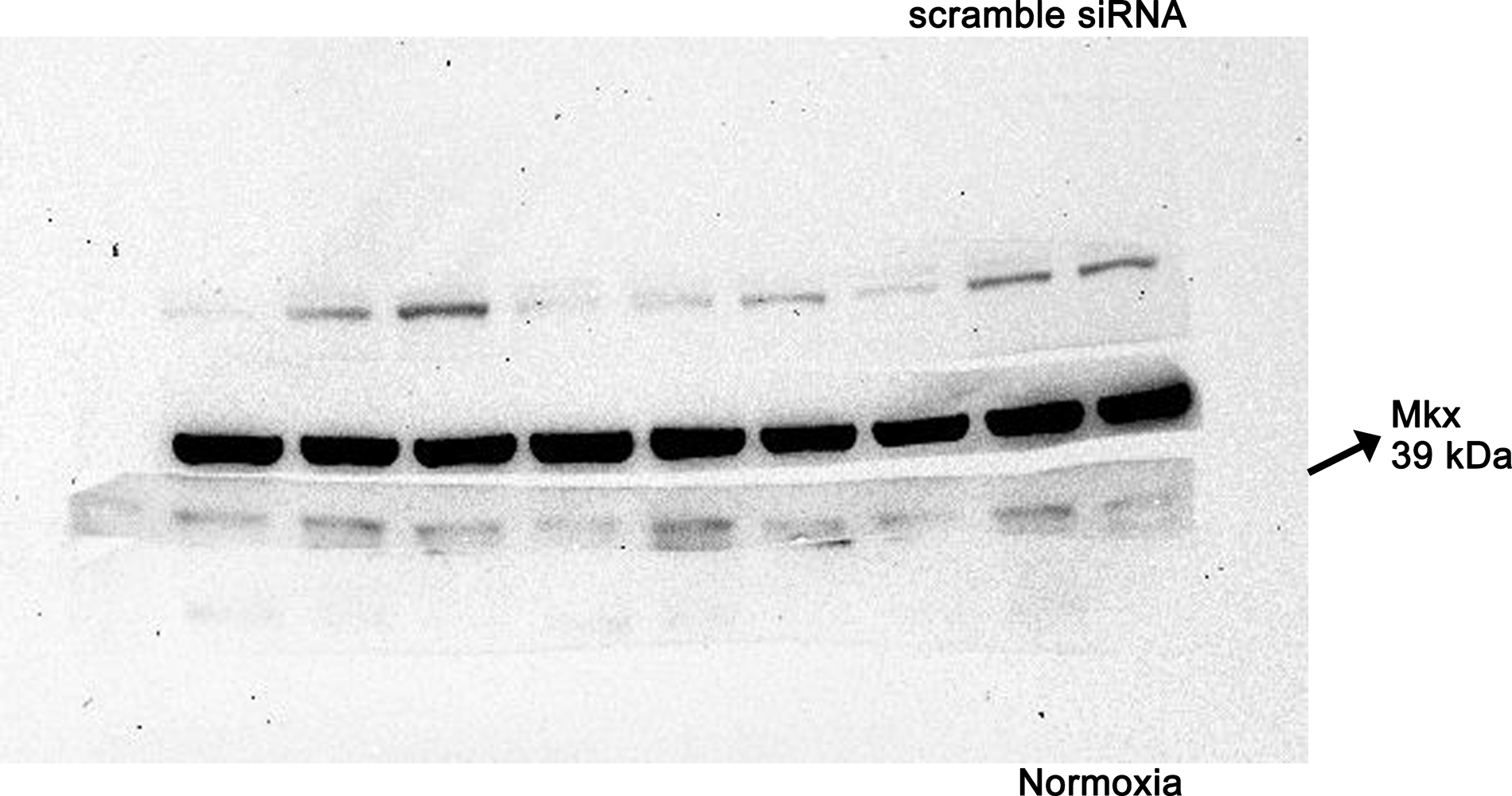

Supplement: Supplementary file 7 — Additional file 7: Supplementary Figure 7: Western blot of Mkx under normoxia condition. [file 13287_2021_2506_MOESM7_ESM.tif]

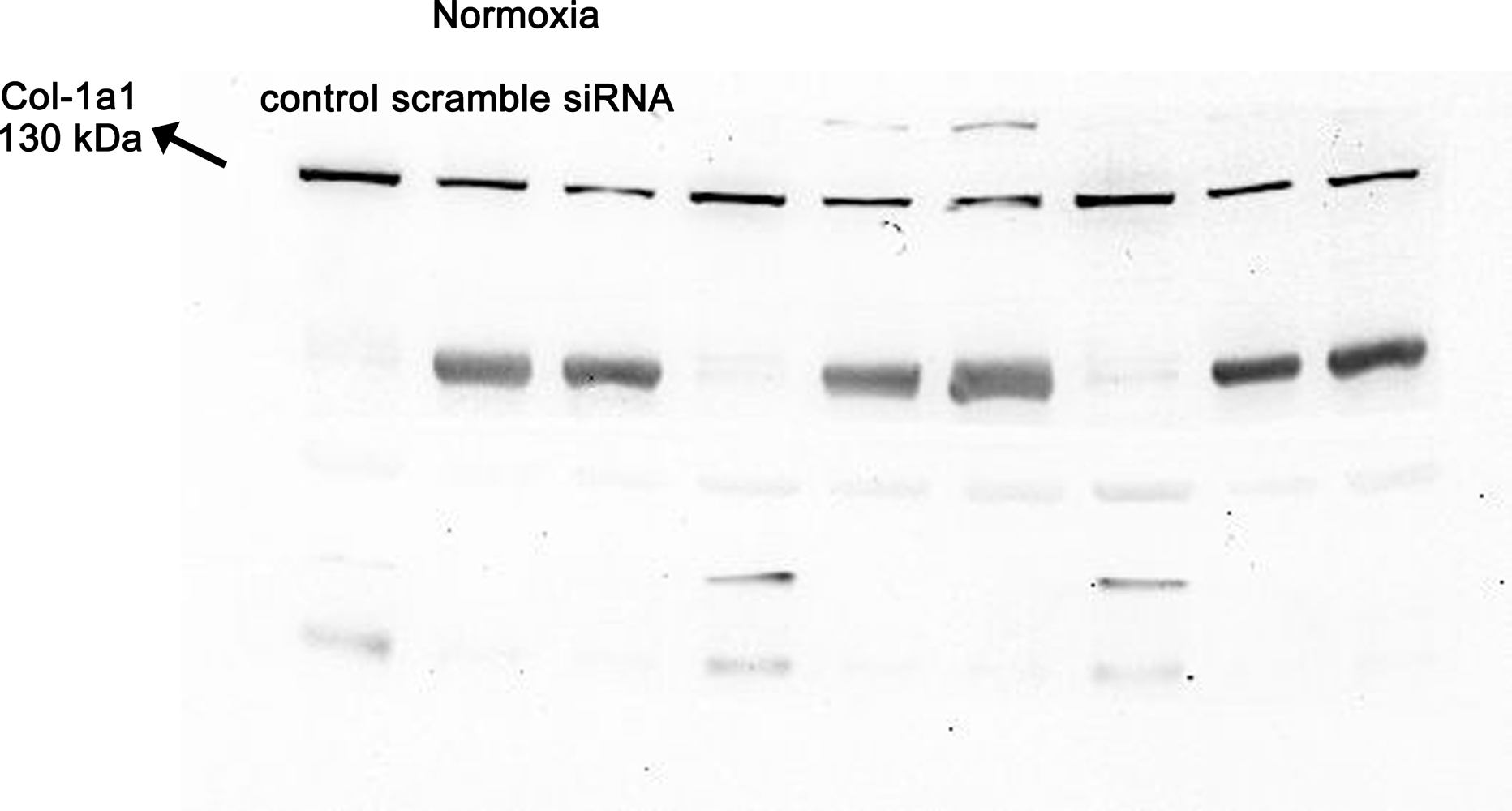

Supplement: Supplementary file 8 — Additional file 8: Supplementary Figure 8: Western blot of Col-1a1 under normoxia condition. [file 13287_2021_2506_MOESM8_ESM.tif]

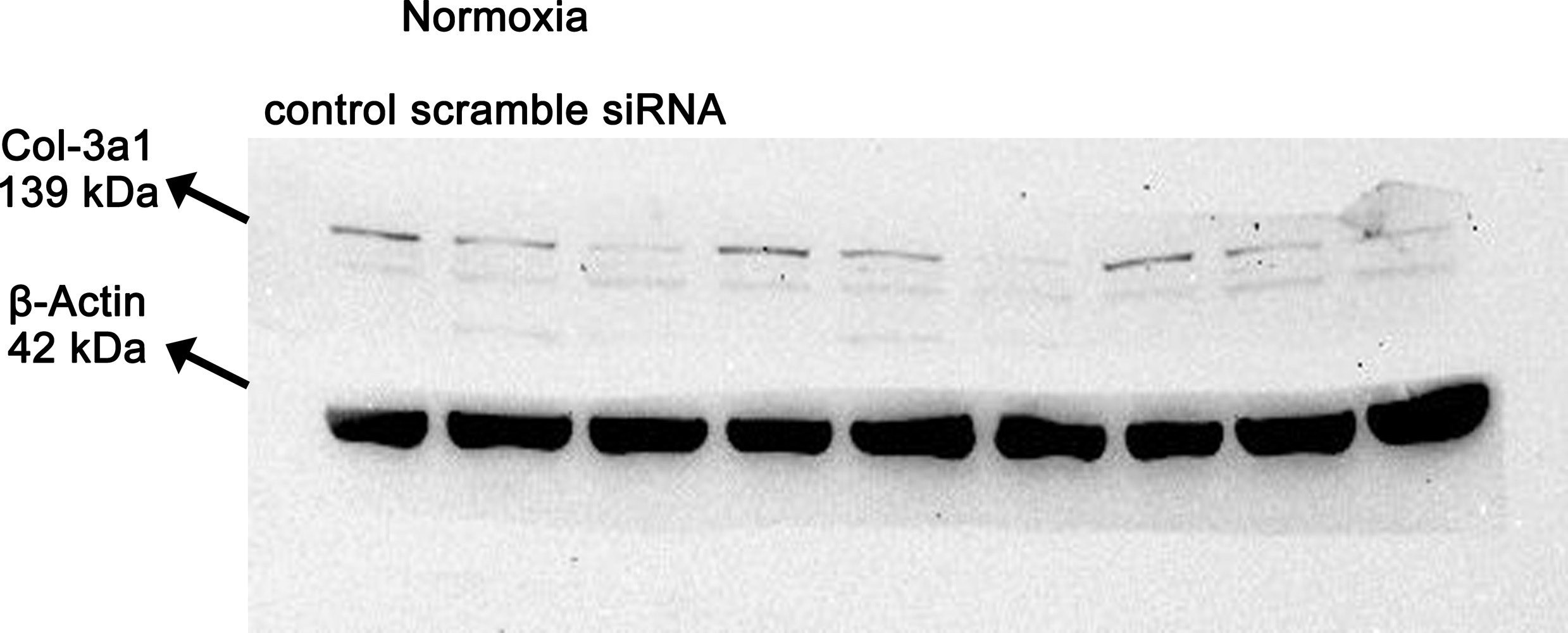

Supplement: Supplementary file 9 — Additional file 9: Supplementary Figure 9: Western blots of Col-3a1 and β-Actin under normoxia condition. [file 13287_2021_2506_MOESM9_ESM.tif]

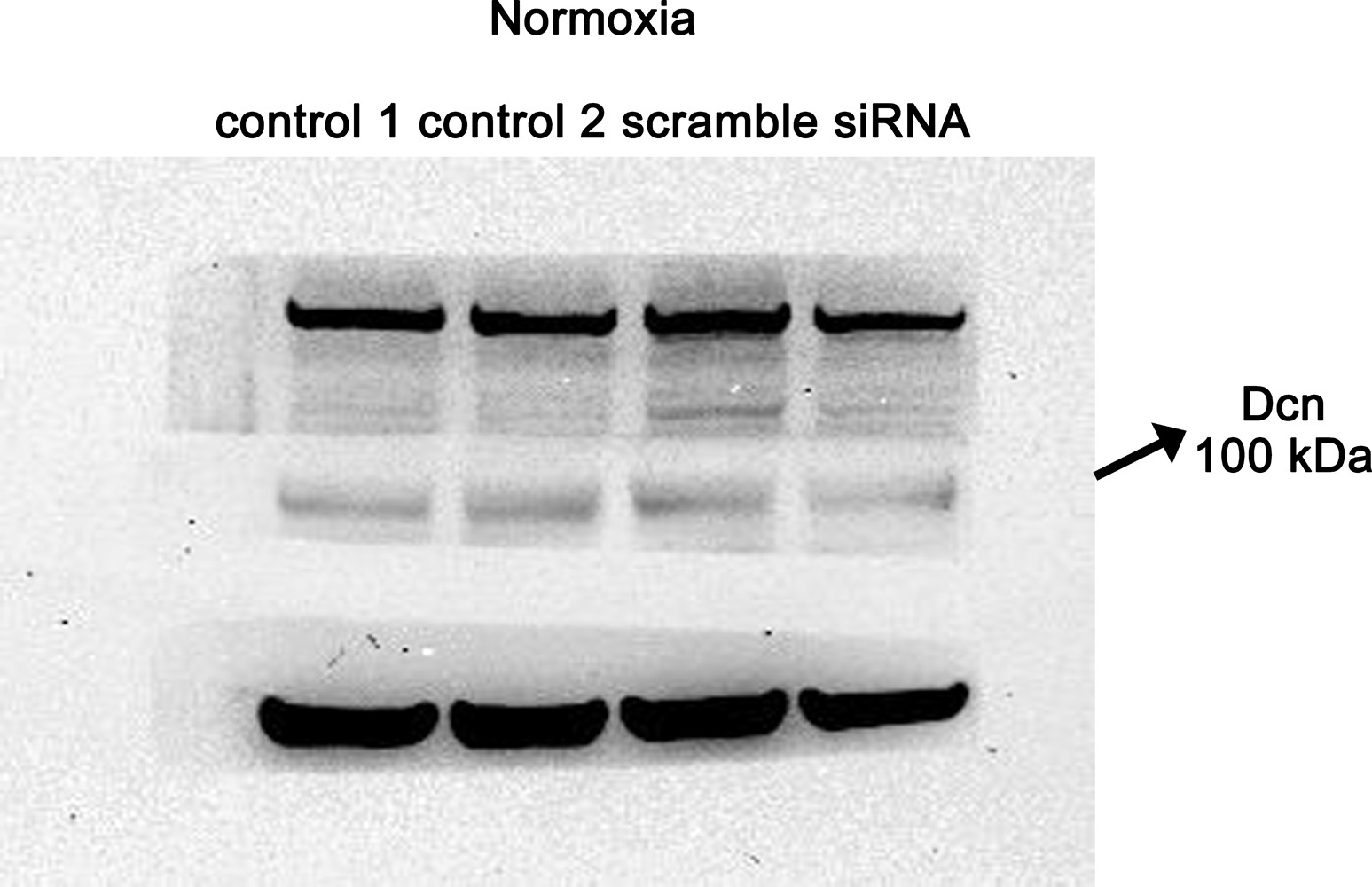

Supplement: Supplementary file 10 — Additional file 10: Supplementary Figure 10: Western blot of Dcn under normoxia condition. [file 13287_2021_2506_MOESM10_ESM.tif]

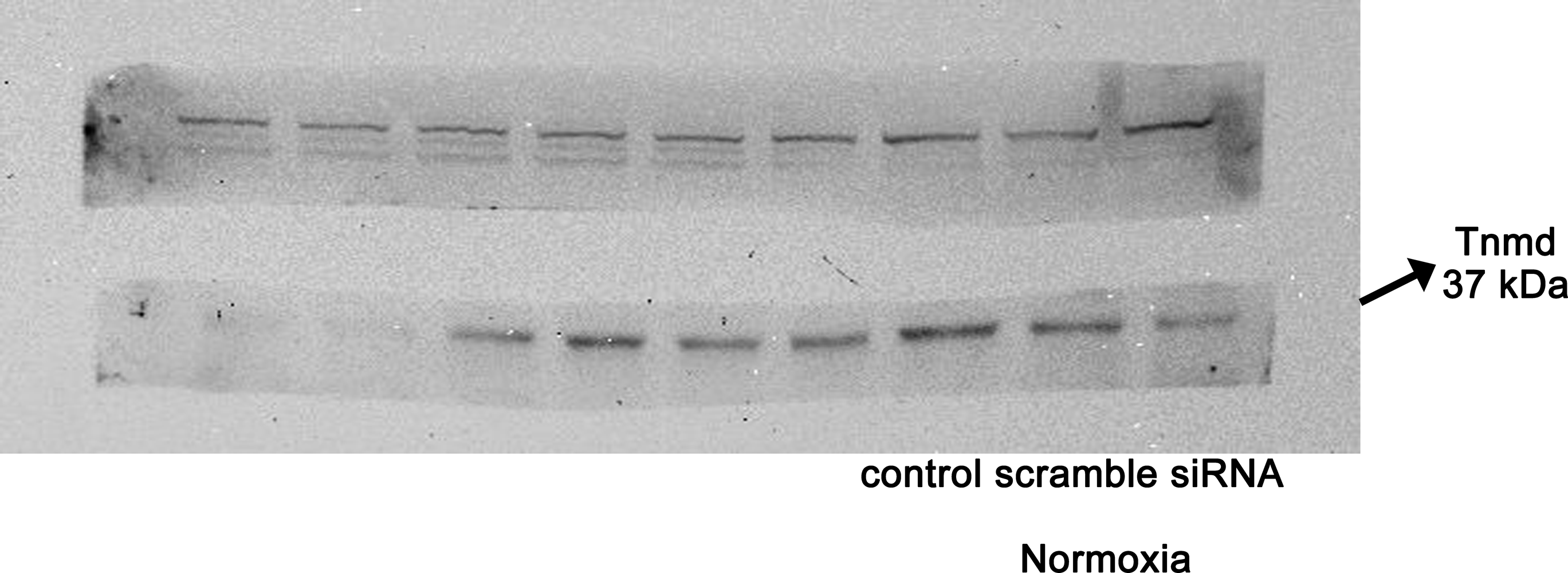

Supplement: Supplementary file 11 — Additional file 11: Supplementary Figure 11: Western blot of Tnmd under normoxia condition. [file 13287_2021_2506_MOESM11_ESM.tif]

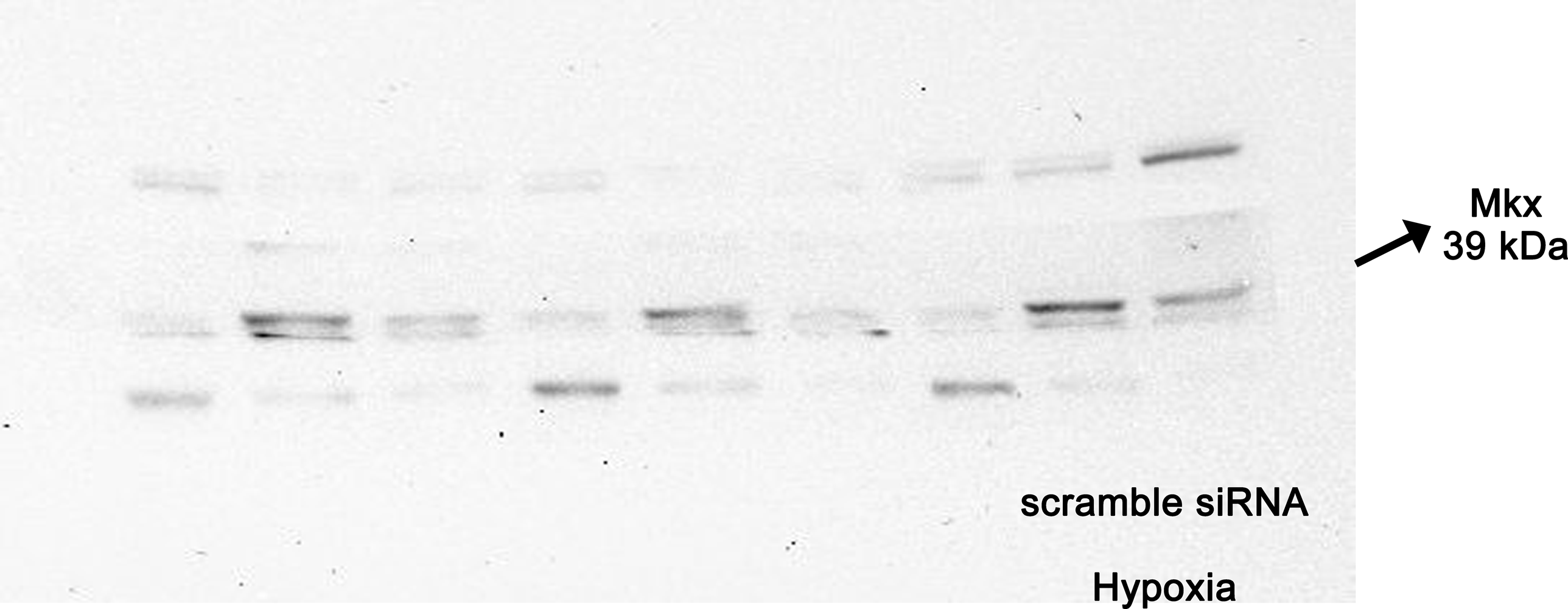

Supplement: Supplementary file 12 — Additional file 12: Supplementary Figure 12: Western blot of Mkx under hypoxia condition. [file 13287_2021_2506_MOESM12_ESM.tif]

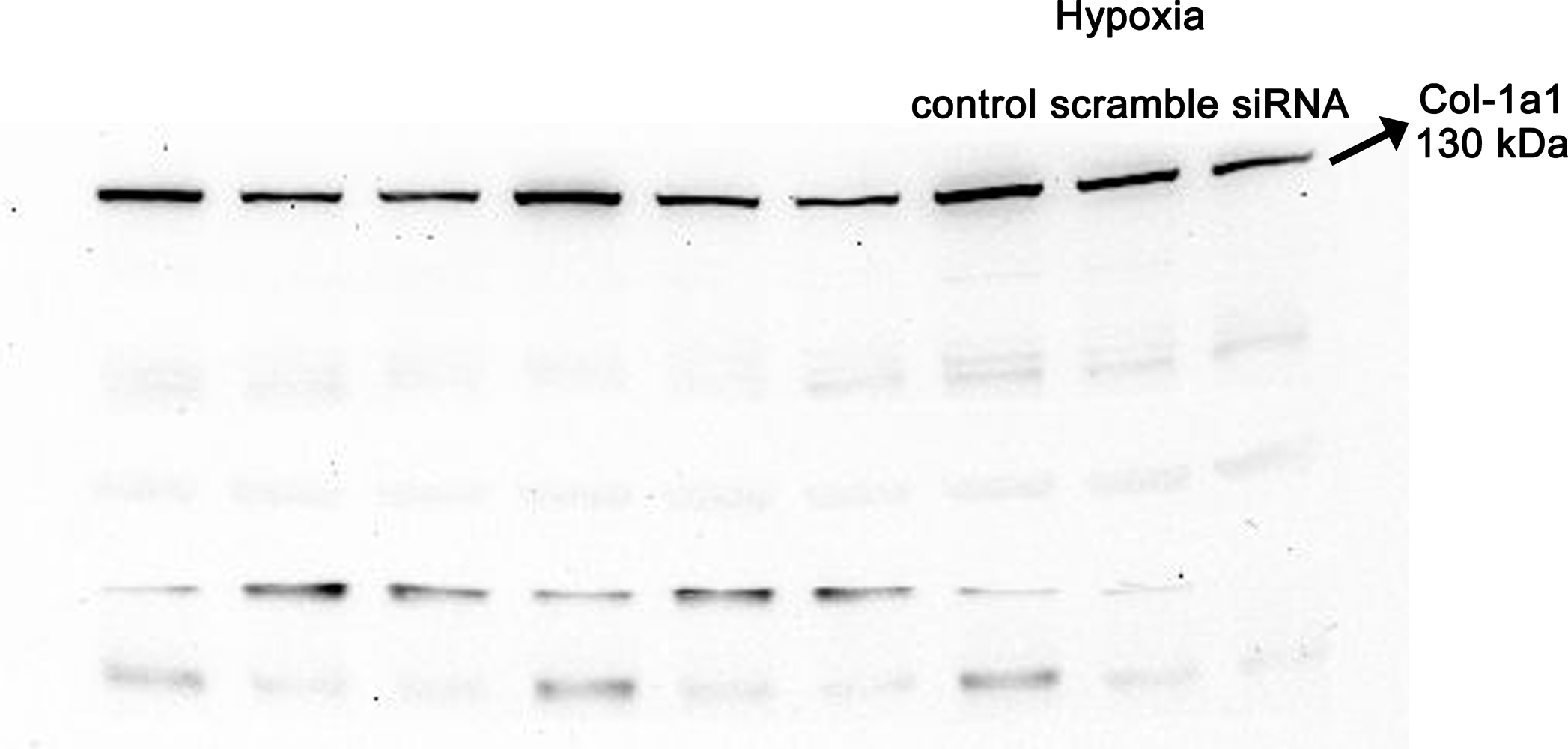

Supplement: Supplementary file 13 — Additional file 13: Supplementary Figure 13: Western blot of Col-1a1 under hypoxia condition. [file 13287_2021_2506_MOESM13_ESM.tif]

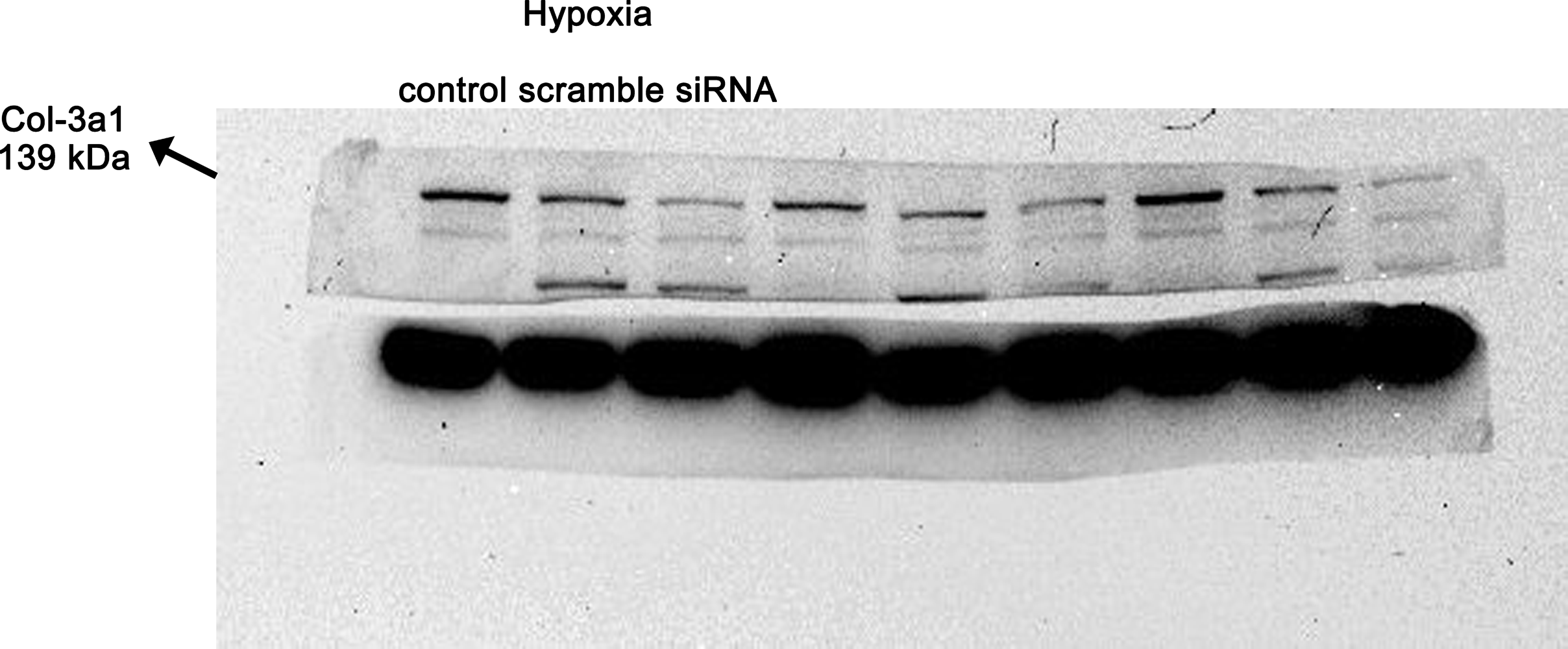

Supplement: Supplementary file 14 — Additional file 14: Supplementary Figure 14: Western blot of Col-3a1 under hypoxia condition. [file 13287_2021_2506_MOESM14_ESM.tif]

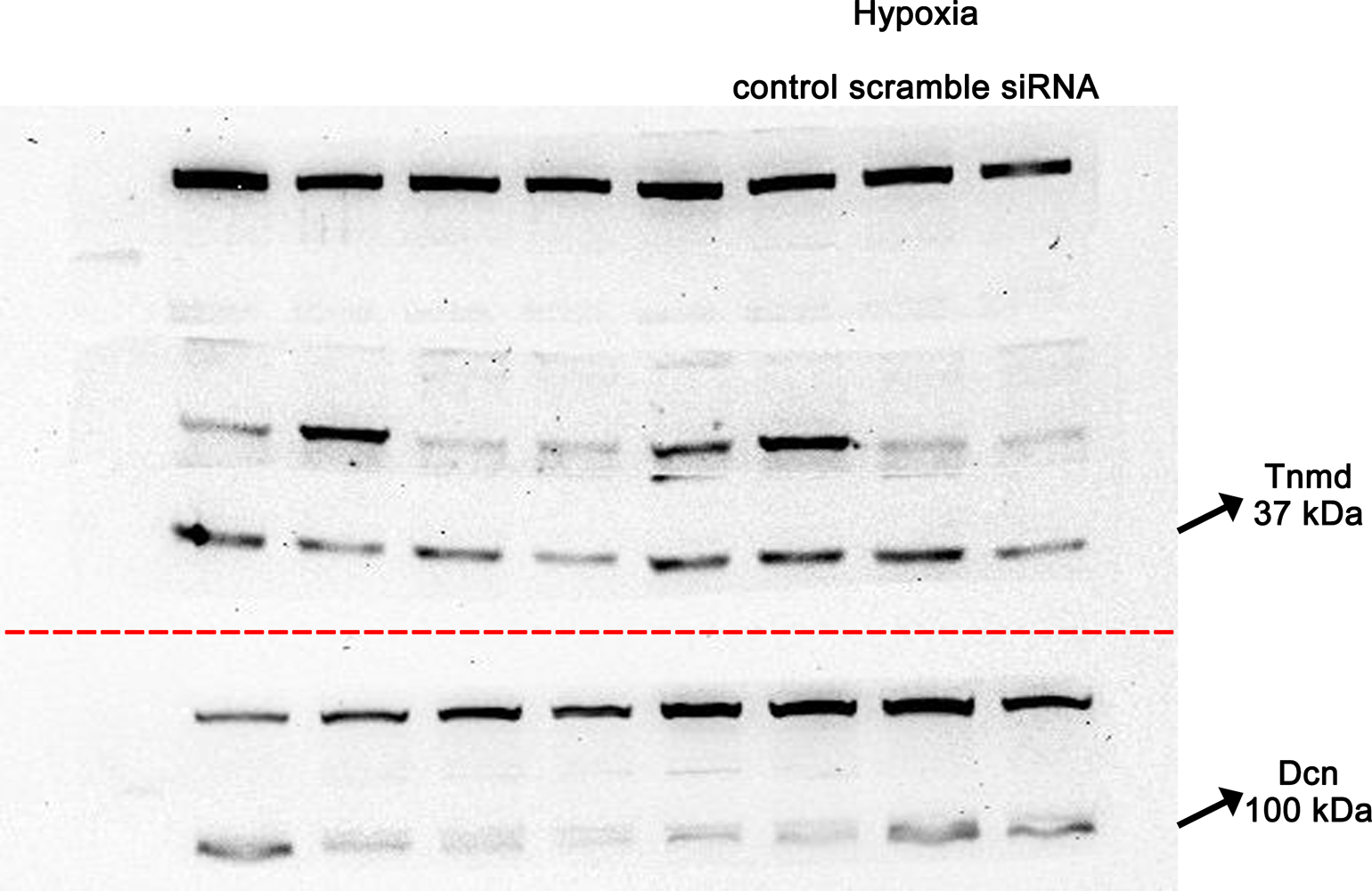

Supplement: Supplementary file 15 — Additional file 15: Supplementary Figure 15: Western blots of Dcn and Tnmd under hypoxia condition. [file 13287_2021_2506_MOESM15_ESM.tif]

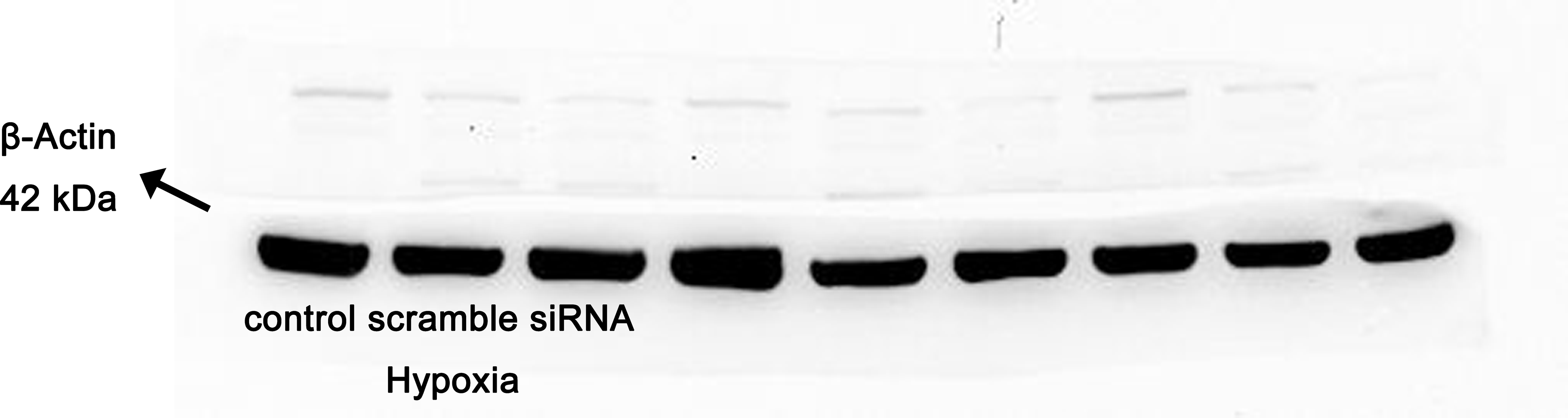

Supplement: Supplementary file 16 — Additional file 16: Supplementary Figure 16: Western blots of β-Actin under hypoxia condition. [file 13287_2021_2506_MOESM16_ESM.tif]

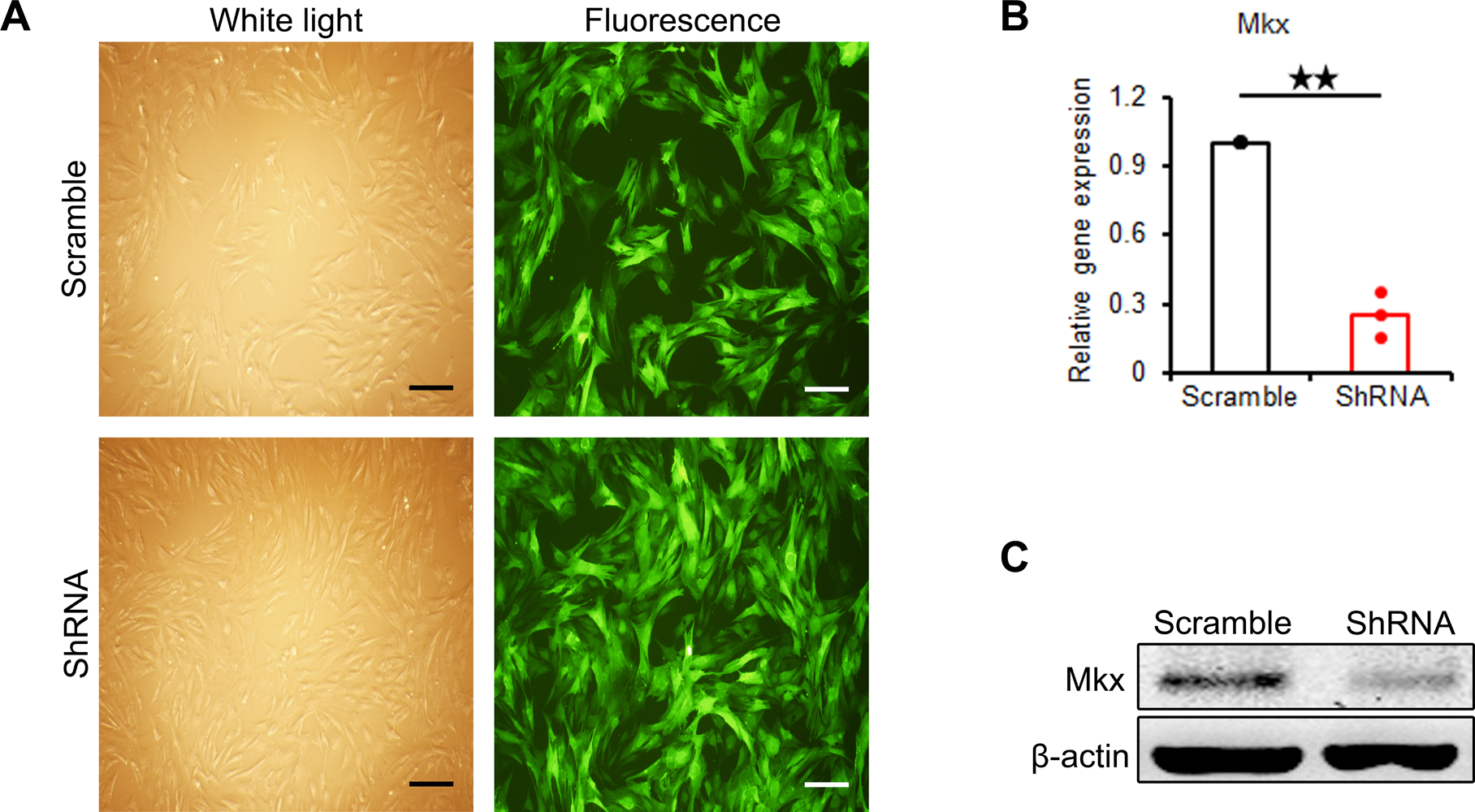

Supplement: Supplementary file 17 — Additional file 17: Supplementary Figure 17: The transfection efficiency of Mkx-specific shRNA in BMSCs. [file 13287_2021_2506_MOESM17_ESM.tif]

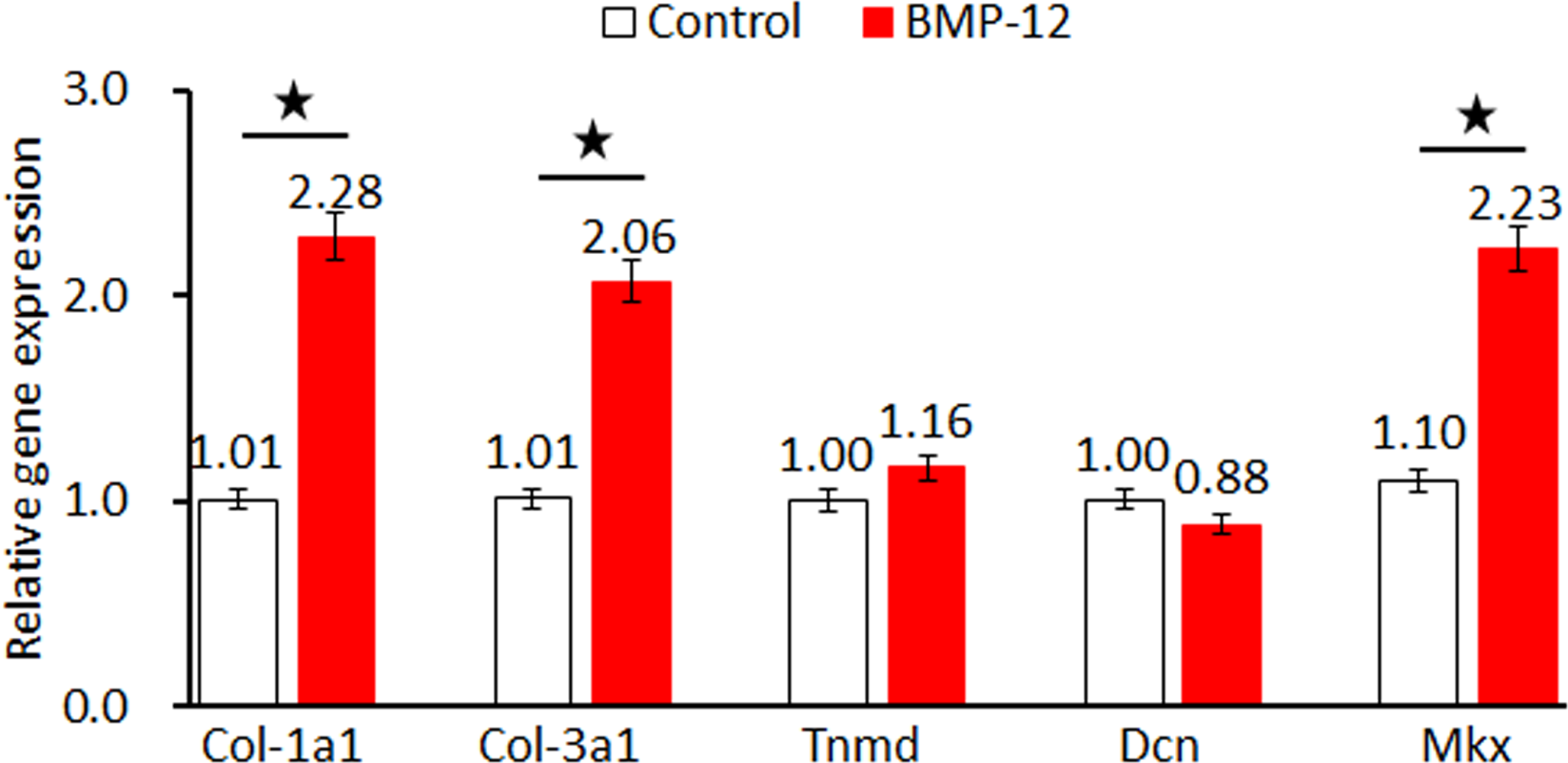

Supplement: Supplementary file 18 — Additional file 18: Supplementary Figure 18: Gene expression of Mkx and tenogenic differentiation markers under BMP-12 condition in BMSCs. [file 13287_2021_2506_MOESM18_ESM.tif]
