# Supplementary material for: Mkx mediates tenogenic differentiation but incompletely inhibits the proliferation of hypoxic MSCs
Source: Stem Cell Res Ther. 2021 Jul 28;12:426. doi: 10.1186/s13287-021-02506-3 (PMC8317301; doi:10.1186/s13287-021-02506-3)
Supplement: Supplementary file 19 — Additional file 19: Supplementary Table 1: Comparison of the biomechanical properties of the hypoxia and shRNA group, the hypoxia and scramble group and the normal control groups in patellar tendon. [file 13287_2021_2506_MOESM19_ESM.docx]

Supplementary table 1. Comparison of the biomechanical properties of the hypoxia and shRNA group, the hypoxia and scramble group and the normal control groups in patellar tendon. (mean ± standard deviation).

|  | Maximum Load (N) | Stiffness (N/mm) | Maximum Stress (N/mm^2^) | Cross-sectional Area (mm^2^) | Elastic Modulus (Mpa) |
| --- | --- | --- | --- | --- | --- |
| Patellar tendon | ☆△ | ☆△ | ☆△ | △ | ☆△ |
| Hypoxia+shRNA | 104.6±40.1  △ | 12.7±7.1  △ | 6.6±1.7  △ | 17.1±1.6  △ | 13±5.9  △ |
| Hypoxia+scramble | 206±16.1 | 30.4±6.5 | 12.9±1.5 | 16±1.4 | 30.9±3.6 |
| Normal control | 295.5 ±28.5 | 41.3±3.8 | 25.7±2.8 | 11.6±1.5 | 61.8±8.7 |

☆Significantly different from the hpoxia+scramble group.

△Significantly different from the normal control group.

☆*p* <0.05; △*p*<0.05.
